# Analysis of key nodes and metabolic pathways in the protein network of secondary metabolism in *Panax quinquefolius* L. enhanced by arbuscular mycorrhizal fungi

**DOI:** 10.1186/s12870-026-08629-0

**Published:** 2026-04-01

**Authors:** Yue Wang, Zhifang Ran, Siqi Ma, Yuting Yao, Ziqi Liu, Ruzhen Wang, Peng Zhang, Lanping Guo, Lei Fang, Jie Zhou

**Affiliations:** 1 School of Biological Science and Technology, University of Jinan, Jinan, 250022 P.R. China; 2 Shandong Academy of Traditional Chinese Medicine, Institute of Chinese Materia Medica Resources, Jinan, 250013 P.R. China; 3 Shandong Zhongping Pharmaceutical Co. Ltd, Linyi , 273399 P.R. China; 4 State Key Laboratory for Quality Ensurance and Sustainable Use of Dao-di Herbs, Beijing, 100700 P.R. China

**Keywords:** Arbuscular mycorrhizal fungi, Panax quinquefolius, Proteomics, Fungal preparation applications

## Abstract

**Background:**

Arbuscular Mycorrhizal Fungi (AMF) formed symbiotic relationships with roots and were capable of promoting the growth of the medicinal plant *Panax quinquefolius* L. as well as the accumulation of its active component, ginsenosides. However, the regulatory mechanisms underlying this process remained unclear. To elucidate the protein signaling pathways through which AMF regulates the secondary metabolism of *P. quinquefolius* and to promote the application of AMF inoculants in the cultivation of medicinal herbs, this study employed a controlled pot experiment, establishing an AMF-inoculated group along with a control group. Tandem mass tag (TMT) labeling quantitative techniques were utilized for the proteomic analysis of the roots, and these results were utilized to conduct a correlation analysis with the previous transcriptomics and metabolomics data.

**Results:**

AMF significantly regulated the growth and protein expression profile of *P. quinquefolius*, leading to the identification of 214 differentially expressed proteins (DEPs). Gene Ontology (GO) functional enrichment analysis indicated that the DEPs were involved in oxidoreductase activity, ligase activity. Kyoto Encyclopedia of Genes and Genomes (KEGG) functional enrichment analysis showed that pathways related to nitrogen metabolism biosynthesis, carbon fixation in photosynthetic organisms, phenylpropanoid biosynthesis, and pyruvate metabolism were significantly enriched among the DEPs. Additionally, pyruvate kinase was identified as a key network node in protein interactions. Multi-omics analysis revealed that proteins such as S-adenosylmethionine synthetase, involved in cysteine and methionine metabolism, and genes such as CYCD3, related to plant hormone signal transduction, were significantly upregulated, with their expression levels showing a significant positive correlation with ginsenoside accumulation.

**Conclusions:**

This study identifies critical nodes and pathways at the protein level through which AMF regulates secondary metabolism in *P. quinquefolius*, providing foundational data for the expansion of AMF research and application in the field of medicinal herbs.

**Supplementary Information:**

The online version contains supplementary material available at 10.1186/s12870-026-08629-0.

## Introduction

Arbuscular mycorrhizal fungi (AMF) are an important group of beneficial microorganisms in soil ecosystems, capable of forming mutualistic symbioses with over 70% of terrestrial plant roots. They assist plants in nutrient acquisition and promote plant growth, making them widely applicable in the agricultural field [1–3]. Studies have shown that AMF can enhance photosynthesis and nutrient metabolism, leading to increased biomass accumulation in the shoots and roots of crops such as maize, sorghum and cotton [4, 5]. Inoculation with AMF improved wheat’s phosphorus uptake, significantly increasing wheat grain yields [6]. Moreover, AMF was found to enhance crop resistance to biotic and abiotic stresses. Research indicated that AMF inoculation significantly boosted the activity of defensive enzymes in carrots, enhancing their resistance to nematodes [7]. Xu et al. [8] reported that AMF inoculation markedly increased the drought and salinity resistance of fruit trees. AMF can serve as an environmentally friendly biofertilizer, improving crop quality and promoting sustainable agricultural development [9]. Shafiq et al. [10] found that in a tomato-onion intercropping system, the combined use of AMF with growth substrates significantly enhanced the accumulation of chlorophyll, iron, and manganese in tomatoes, increasing their nutritional value. Thus, AMF demonstrated active roles in promoting crop growth, improving stress resistance, and enhancing quality, making them an important biotechnological tool for advancing sustainable agriculture.

The cultivation of traditional Chinese medicinal materials, as an important branch of modern agricultural development, plays an indispensable strategic role in promoting the optimization and upgrading of agricultural industry structure and ensuring the sustainable development of traditional Chinese medicine. Studies have shown that AMF can induce the accumulation of active substances in medicinal herbs such as *Atractylodes macrocephala* and *Salvia miltiorrhiza*, thereby enhancing the quality of these herbs [11, 12]. Investigating the mechanisms by which AMF influence the formation of medicinal herb quality is of significant importance for expanding their application in the production of traditional Chinese medicinal materials. Its mechanism of action is characterized by multi-level and synergistic regulation. At the nutritional and physiological level, AMF enhanced the host’s absorption of mineral nutrients such as phosphorus and nitrogen, promoting the synthesis of secondary metabolites [13]. At the level of transcriptional regulation, AMF colonization significantly upregulated the expression of key enzyme genes in the phenylpropanoid pathway, such as phenylalanine ammonia-lyase (PAL) and chalcone synthase (CHS), facilitating the biosynthesis and accumulation of bioactive compounds such as flavonoids and terpenes [14]. In terms of signal transduction and hormone regulation, AMF colonization induced the production of signals such as nitric oxide (NO) and hydrogen peroxide (H_2_O_2_) within the plant, and regulated the levels of hormones like jasmonic acid (JA). This occured through the activation of transcription factors from the MYB and WRKY families, initiating transcriptional reprogramming of downstream genes related to secondary metabolism [15]. In summary, AMF drove the biosynthesis of secondary metabolites in medicinal plants efficiently through the integration of nutritional improvement, signal activation, and transcriptional regulation at the transcriptomic and metabolic functional levels.

Proteins are the final products of gene expression, and research on protein expression levels and post-translational modifications can directly reflect cellular metabolism and phenotypic characteristics [16]. Currently, research on the mechanisms of AMF at the protein level in the production of traditional Chinese medicinal materials, particularly in the field of precious medicinal herbs, is still relatively scarce. *Panax quinquefolius* L., commonly known as American ginseng, is a precious medicinal herb recognized for its functions in tonifying qi, nourishing yin, clearing heat, and generating fluids, with over 300 years of medicinal history in China [17, 18]. *P. quinquefolius* is rich in various effective components such as ginsenosides, polysaccharides, and flavonoids [19–21], and possesses pharmacological activities such as anti-tumor and anti-aging effects [22–25]. Ginsenosides are the most significant active compounds contributing to the pharmacological effects of *P. quinquefolius* and serve as a key indicator for evaluating the quality of this medicinal herb [26]. Our research group has previously discovered abundant AMF resources in the roots of *P. quinquefolius* and, through transcriptomics and metabolomics approaches, revealed the mechanisms by which AMF promote the growth and secondary metabolism of *P. quinquefolius* at the gene expression level [27–30]. However, given that proteins are the direct executors of function, identifying the key proteins and pathways responsive to AMF from a proteomic perspective was an important entry point for analyzing the mechanisms underlying the formation of active constituents in *P. quinquefolius*. This approach held significant importance in addressing the research gap at the protein level within this field.

Based on previous research, this study conducted a controlled pot experiment by inoculating *P. quinquefolius* seeds with the arbuscular mycorrhizal fungus *Rhizophagus intraradices*, and employed Tandem Mass Tag (TMT) labeling quantitative technology for proteomic analysis of the plant root tissues. The objectives of this study were as follows: (1) to clarify the effects of AMF inoculation on the biomass of *P. quinquefolius* seedlings and assess its growth-promoting effects; (2) to identify the key proteins and metabolic pathways involved in the regulation of metabolite accumulation in *P. quinquefolius* by AMF based on differential protein expression, revealing the molecular regulatory mechanisms; (3) to integrate findings with previous transcriptomic and metabolomic data to elucidate the secondary metabolic pathways mediated by AMF in *P. quinquefolius*. This study clarified the key nodes and pathways by which AMF regulates the accumulation of metabolites in *P. quinquefolius* at the protein level, providing foundational data to expand the research and application of AMF in the field of traditional Chinese medicinal materials.

## Materials and methods

### Experimental design and sample collection

The AMF strain used in this study is *Rhizophagus intraradices* (BEG YN09; formerly known as *Glomus intraradices*), which was purchased from the Institute of Plant Nutrition and Resources, Beijing Academy of Agricultural and Forestry Sciences. The inoculum consists of mycelium, spores (approximately 50 spores per gram of inoculum), host root segments, and culture substrate. *Panax quinquefolius* seeds were collected from a cultivation base in Wendeng District, Weihai City, Shandong Province. The seeds were surface sterilized by soaking in a 2% NaHClO solution for 30 min, followed by thorough rinsing with sterile water three times. After soaking for 24 h, the seeds were germinated in an incubator at 25 °C [31]. Select sandy loam soil with a preceding crop of wheat and other crops. The soil is also sourced from the *P. quinquefolius* cultivation base in the Wendeng District of Shandong Province, characterized by a pH of approximately 6.5, effective phosphorus content of 77.49 mg/kg, effective nitrogen content of 105.61 mg/kg, effective potassium content of 38.04 mg/kg, and organic matter content of 1.61 g/kg.

A controlled pot experiment was conducted with the following treatments: a control group (non-AMF) and an AMF group. Each treatment comprised three replicates, with eight pots per replicate, resulting in a total of 48 pots. The culture substrate was prepared by mixing soil and sand in a 2:1 (v/v) ratio, which was sterilized by autoclaving at 121 °C and 0.10 MPa for 2 h to ensure the elimination of any existing AMF propagules and other microorganisms. Each pot was filled with 2 kg of sterilized substrate, into which uniform, disease-free *P. quinquefolius* seeds were sown, with 15 seeds planted per pot.In the AMF treatment group, 10% (v/v) of *R. irregularis* was evenly applied to a depth of 1.5 cm below the seeds, while the control group received an equivalent amount of autoclaved mycorrhizal inoculum to maintain consistent substrate composition across treatments. All pots were placed under greenhouse conditions set to a day/night temperature of 25/20°C, with relative humidity at 70/65%, and a photoperiod of 16/8 hours, providing a photosynthetic photon flux density (PPFD) of 100 µmol m^−2^s^− 1^, with fluorescent lights as the source. Water was applied as needed to maintain normal growth conditions for *P. quinquefolius*. The positions of the pots were randomly adjusted weekly to minimize environmental variation. All pots were irrigated with 100 ml of a diluted Hoagland solution at a strength of 1/10 P (0.1 mM). After 12 weeks of treatment, six randomly selected central plants from each group were measured for growth indicators. Fresh roots of *P. quinquefolius* were collected and divided into two parts: the main roots and the fibrous roots. The main roots from each replicate, which included eight potted plants, were thoroughly mixed, with three replicates for each treatment, and stored at -80 °C for proteomic analysis. The fibrous roots were used to determine mycorrhizal colonization.

### Determination of AMF colonization rate

The roots of *P. quinquefolius* were washed with distilled water and cut into approximately 1 cm long segments, which were then placed into test tubes containing a 10% KOH solution. The samples were heated in a water bath at 90 °C for 40 min. After six washes with distilled water, 2% HCl solution was added to the test tubes, and the samples were soaked at room temperature for 5 min before discarding the liquid. Subsequently, the root samples were stained with a 0.05% trypan blue solution to enhance the visibility of fungal structures. According to the methods described by Yang et al. [32] and Ouledali et al. [33], the level of mycorrhizal colonization was assessed using a fluorescence microscope (Olympus bx51) under bright-field conditions (the magnification of the objective lens ranges from 10× to 40×). If typical structures such as hyphae, arbuscules, or vesicles are observed, it indicates that AMF has successfully colonized the *P. quinquefolius* root system [21]. Each slide contains 10 root segments, and each treatment group has 6 replicates, resulting in 6 slides. The AMF colonization rate (%) was calculated as follows: AMF colonization rate (%) = (number of colonized root segments / total number of observed root segments) × 100%.

### Protein extraction and quality assessment

Fresh *P. quinquefolius* roots were cryo-ground and lysed in SDT buffer (4% SDS, 100 mM DTT, 10 mM TEAB) with sonication on ice. Following centrifugation (12,000 g, 4 °C, 15 min), the supernatant was sequentially reduced (56 °C, 1 h) with 10 mM DTT and alkylated in the dark with iodoacetamide (1 h). Proteins were precipitated with four volumes of ice-cold acetone (≥ 2 h, -20 °C), pelleted by centrifugation, and washed thrice with cold acetone. The final protein pellet was solubilized in buffer (8 M urea, 100 mM TEAB, pH 8.0).

Protein concentrations were determined using the Bradford protein quantification kit (Bio-Rad Laboratories, Hercules, CA, USA). BSA standards and diluted samples were added to a 96-well plate in triplicate, with the volume adjusted to 20 µL. After adding 180 µL of G250 dye and incubating for 5 min at room temperature, absorbance was measured at 595 nm. Sample concentrations were calculated from a standard curve. For each sample, 20 µg of protein was separated on a 12% SDS-PAGE gel (80 V for 20 min, then 120 V for 90 min), followed by Coomassie Brilliant Blue R-250 staining and destaining until bands were clear.

### Protein digestion and Tandem Mass Tag (TMT) labeling

During the trypsin digestion, 100 µg of protein was extracted from each sample and added to a digestion buffer (100 mM TEAB, pH 8.0) containing 2 µg of Trypsin Gold (Promega, Madison, USA) and incubated overnight at 37 °C. Formic acid was then added to adjust the pH to ≤ 3, mixed, and centrifuged at 12,000 g for 5 min at room temperature. Peptides were desalted using a C18 chromatographic column and then vacuum freeze-dried. The dried peptides were reconstituted in 100 µL of 0.1 M TEAB buffer and labeled according to the instructions of the TMT kit (Thermo Fisher Scientific). Peptide samples were separated at high pH (pH = 10) by increasing the ACN concentration using a Waters BEH C18 chromatographic column (4.6 × 250 mm, 5 μm) on an L-3000 HPLC system (Thermo DINOEX, USA).

### Liquid chromatography-mass spectrometry (LC-MS/MS) analysis

Peptides were dissolved in mobile phase A (0.1% formic acid, 2% acetonitrile) and analyzed via LC-MS/MS using an EASY-nLC™ 1200 UHPLC coupled to a Q Exactive™ HF-X mass spectrometer equipped with a Nanospray Flex™ source. Peptide separation (600 nL/min) employed a 90-min linear gradient (6-100% mobile phase B: 0.1% formic acid in 80% acetonitrile) on a 15 cm × 150 μm homemade column (1.9 μm). The gradient was as follows: 6–15% B (2 min), 15–40% B (76.5 min), 40–50% B (2 min), 50–55% B (1 min), and 100% B (8.5 min). Full MS scans (350–1500 m/z) were acquired at 60,000 resolution, with HCD MS/MS analysis of the top 40 most abundant precursors.

### Protein identification and quantification, and the screening of differentially expressed proteins (DEPs)

The raw spectral data obtained from mass spectrometry were analyzed using Proteome Discoverer 2.2 software, along with filtering based on the RNA-Seq database (https://submit.ncbi.nlm.nih.gov/subs/bioproject/). The search parameters for Proteome Discoverer 2.2 were set as follows: maximum allowable peptide mass deviation of ± 10 ppm; mass tolerance for product ions of ± 0.05 Da, with a total protein false discovery rate (FDR) of 1%. Statistical analysis of the protein quantification results was performed using a T-test, and the fold change in protein levels (> 1.2 or < 0.83, *P* < 0.05) was used as a screening criterion for differentially expressed proteins (DEPs) between the AMF group and the control group [34]. To eliminate interference from fungal proteins, rigorous filtering was conducted during data analysis. The obtained spectral data were matched against both plant and fungal databases, categorizing each protein based on peptide sequences into plant proteins, fungal proteins, or shared proteins. Fungal proteins were filtered out, and expression differences between plant proteins in the AMF group and the control group were compared.

### Sources of transcriptomic and metabolomic data

In previous studies, we analyzed the differentially expressed genes (DEGs) and the differentially expressed metabolites (DEMs) of *P. quinquefolius* between the AMF group and the control group (The data has been uploaded to the NCBI database, SRA data: PRJNA868946) (Table S7) [29]. The preliminary joint analysis of transcriptomics and metabolomics [30] serves as the foundational basis for the KEGG association analysis of the differentially expressed genes (DEGs), differentially expressed proteins (DEPs), and differentially expressed metabolites (DEMs) between the AMF inoculation group and the control group in this study.

### Data statistics and analysis

Statistical analysis was performed using R (v4.1.0). Intergroup comparisons were conducted using Student’s t-test, and GraphPad Prism Version 9.5 (San Diego, CA, USA) was utilized to create graphs to clarify the differences in growth metrics between groups. Gene Ontology (GO) functional annotation of the proteins was performed using InterProScan software [35]. To evaluate the metabolic functions of the DEPs identified in the metabolic pathway analysis [36], pathway analysis was conducted using the Kyoto Encyclopedia of Genes and Genomes (KEGG) database. Subsequently, KEGG enrichment analysis was performed to further examine whether the DEPs had significantly enriched functional categories. The subcellular localization of the DEPs was predicted using the Plant-mPLoc server. Protein-protein interactions (PPIs) were analyzed using the STRING database (http://STRING.embl.de/), and diagrams were generated using Cytoscape 3.9.2 to predict the physical and functional interactions among the proteins [37]. Use the corrplot and mixOmics packages in R-4.1.0 to create correlation analysis heatmaps and network diagrams for DEPs, DEGs, and DEMs. Pearson correlation analysis was used to calculate the correlations between proteins, genes, and metabolites. *P*-value < 0.05 was considered to indicate statistical significance.

## Results and analysis

### AMF colonization affects the growth of *P. quinquefolius*

**Fig. 1:**
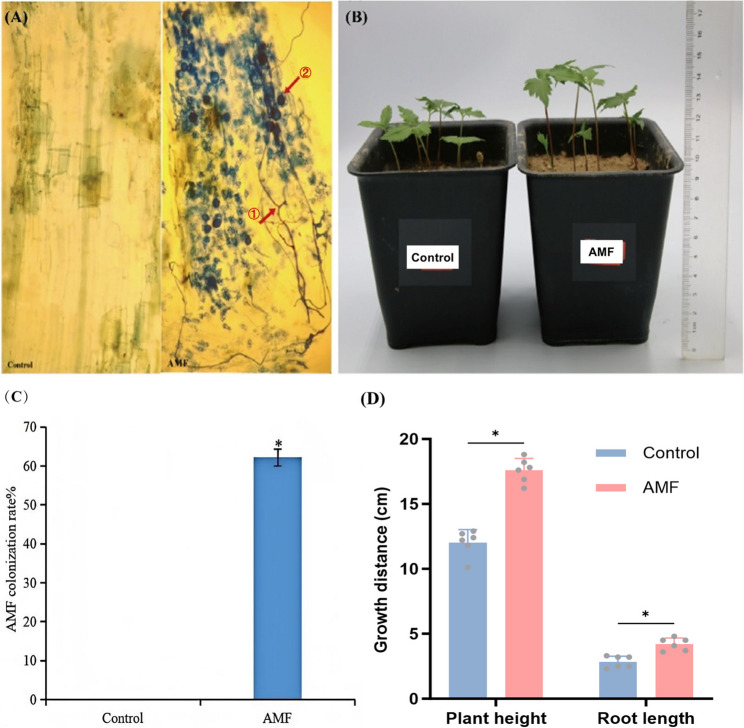
Mycorrhizal morphology and growth status of *P. quinquefolius* roots inoculated (AMF) or not (Control) with *R. irregularis.* **A** The mycorrhizal morphology of *P. quinquefolius* roots with different treatment (①: Arbuscules, ②: Vesicles); **B** Growth status of *P. quinquefolius* with different treatment; **C** Mycorrhizal colonization rate in *P. quiquefolius* roots with different treatment; **D** Growth indicators of *P. quiquefolius* under different treatments. The data were presented as the mean ± SD (*n* = 6). Asterisks indicate significant differences (*P* < 0.05, Student’s t-tests) between the AMF and the control group

To investigate the colonization status of AMF in the roots and its effects on the growth of *P. quinquefolius*, we established AMF-inoculated and non-inoculated groups for *P. quinquefolius* (Fig. 1). Microscopic examination results indicated that no mycorrhizal infection was observed in the root systems of the control group; however, extensive colonization of structures such as arbuscules and vesicles was observed in the root cortex of the AMF-inoculated group, indicating a good mycorrhizal symbiotic relationship with *P. quinquefolius* (Fig. 1A). The colonization rate of AMF in the roots reached 62.21%, in stark contrast to the control group (Fig. 1C). The growth characteristics of *P. quinquefolius* demonstrated that the plant height and root length of AMF-inoculated *P. quinquefolius* was significantly higher than that of the control group (*P* < 0.05) (Fig. 1B, D). These results suggest that AMF can colonize the roots of *P. quinquefolius* after inoculation, forming a beneficial mycorrhizal symbiosis that promotes its growth and development.

### AMF affects the expression of proteins in *P. quinquefolius*

**Fig. 2:**
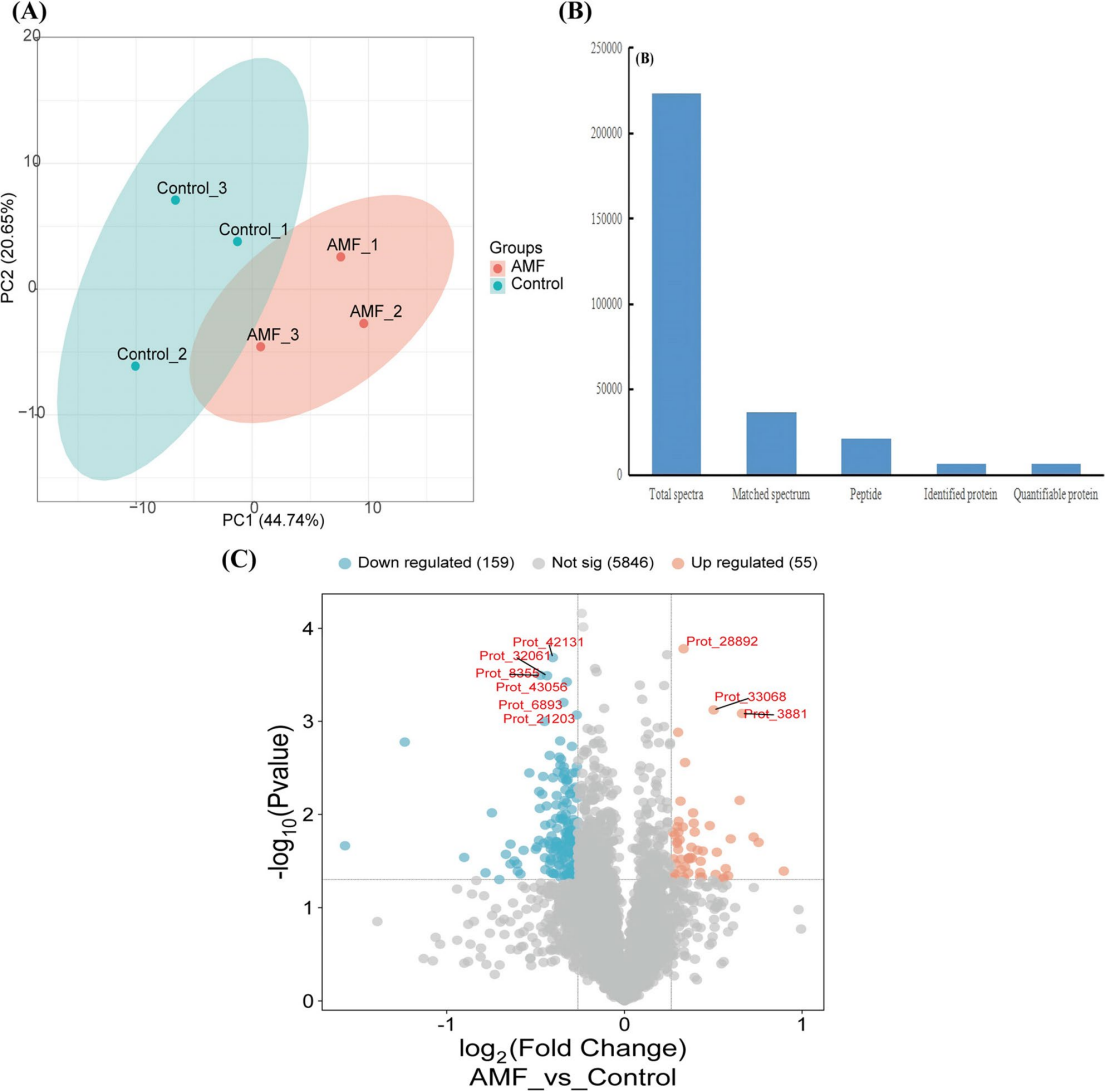
The protein expression profiles and the distribution of differentially expressed proteins (DEPs) between the AMF group and the control group. **A** Principal component analysis between AMF and Control (*n* = 3); **B** Spectrum, peptides and proteins identified from root proteome profiles; **C** Volcano plot of DEPs between AMF and the control group after log transformation. Orange represents upregulated proteins in the AMF treatment group, while blue represents downregulated proteins. The figure highlights the top nine significantly differentially expressed proteins. FC ≥ 1.2 (upregulated) or FC ≤ 0.83 (downregulated), *P* ≤ 0.05

Utilizing TMT-based comparative proteomics, we analyzed the differences in the protein profiles of *P. quinquefolius* between the AMF-inoculated group and the control group. Principal component analysis (PCA) indicated that samples within the same treatment group were spatially clustered, suggesting good reproducibility among the samples in each group. In contrast, there was a significant separation between the groups, indicating notable differences in protein expression among the different treatment groups (Fig. 2A). A total of 223,736 spectra were detected, of which 36,866 spectra matched known protein databases. Additionally, 20,966 unique peptides were identified, leading to the detection of 6,074 specific proteins, of which 6,060 proteins were quantified (Fig. 2B). Most of the identified proteins exhibit good integrity and meet the quality requirements (Figure S1A-C). We screened for differentially expressed proteins (DEPs) based on criteria of FC ≥ 1.2 (upregulated) or FC ≤ 0.83 (downregulated) with *P* ≤ 0.05. The volcano plot reveals significant differences in protein abundance between the AMF group and the control group (Fig. 2C). A total of 214 DEPs were identified, among which 55 DEPs (e.g., Prot_28892) were upregulated and 159 DEPs (e.g., Prot_42131) were downregulated. This indicates that AMF inoculation significantly altered the protein expression profile in *P. quinquefolius*.

### AMF influences the biological functions of differentially expressed proteins in *P. quinquefolius*

**Fig. 3:**
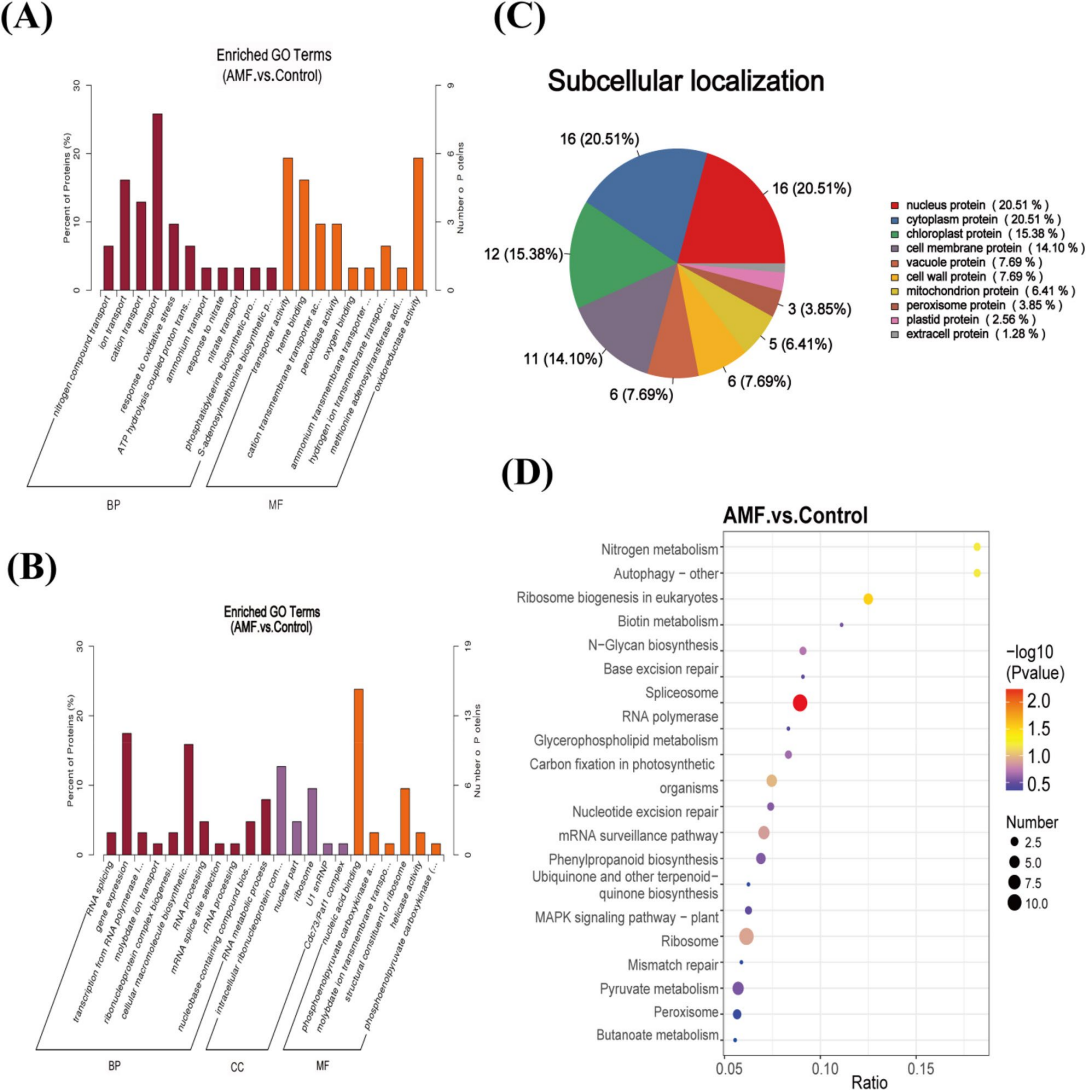
GO enrichment analysis、KEGG enrichment and subcellular localization of DEPs. **A** GO annotation of upregulated DEPs; **B** GO annotation of downregulated DEPs. BP: Biological Process; MF: Molecular Function; CC: Cellular Component; **C** Subcellular classification; **D** KEGG functional enrichment. The protein count refers to the number of DEPs mapped to a specific pathway. P-values were calculated using the hypergeometric test with Bonferroni correction. The enrichment factor represents the ratio of the number of DEPs mapped to a specific pathway to the total number of proteins mapped to that pathway

Through GO analysis, functional clustering was performed on the identified DEPs, resulting in the identification of 138 GO terms, among which 72 represented biological processes, 52 represented molecular functions, and 13 represented cellular components (Table S1). The top 22 GO terms with the highest enrichment levels were selected and categorized (Fig. 3A, B). The results indicated that, compared to the control group, the upregulated proteins in the AMF-inoculated group were significantly enriched in transport, transporter activity and oxidoreductase activity (Fig. 3A, Table S2). These proteins provided a foundation for the synthesis of secondary metabolites. In the downregulated protein group, significant enrichment was observed in the gene expression, nucleic acid binding and cellular macromolecule biosynthetic process (Fig. 3B, Table S2). In summary, these differentially expressed proteins exhibit a variety of molecular functions, and AMF inoculation regulatesd multiple molecular biosynthetic pathways, environmental response mechanisms, enzyme activities, and cellular structures in *P. quinquefolius* roots.

Subcellular localization shows that 214 DEPs are divided into 10 categories (Fig. 3C, Table S3), with the largest proportions found in cytoplasmic proteins (43) and nuclear proteins (43), followed by chloroplast proteins (32) and plasma membrane proteins (30). This indicates that after AMF inoculation, the main differential biological responses in *P. quinquefolius* occur in the cytoplasm, chloroplasts, and nucleus. Functional enrichment analysis showed that DEPs were widely involved in nitrogen metabolism, photosynthetic carbon assimilation, phenylpropanoid metabolism, and pyruvate metabolism processes (Fig. 3D, Table S4). Notably, the enrichment results of these KEGG pathways were consistent with the metabolic pathway changes previously reported by our research group following AMF inoculation in *P. quinquefolius* [29]. This revealed the key role of these pathways in constructing metabolic networks and driving the synthesis of primary and secondary metabolites.

### AMF affects the expression of proteins involved in the metabolic processes of *P. quinquefolius*

**Fig. 4:**
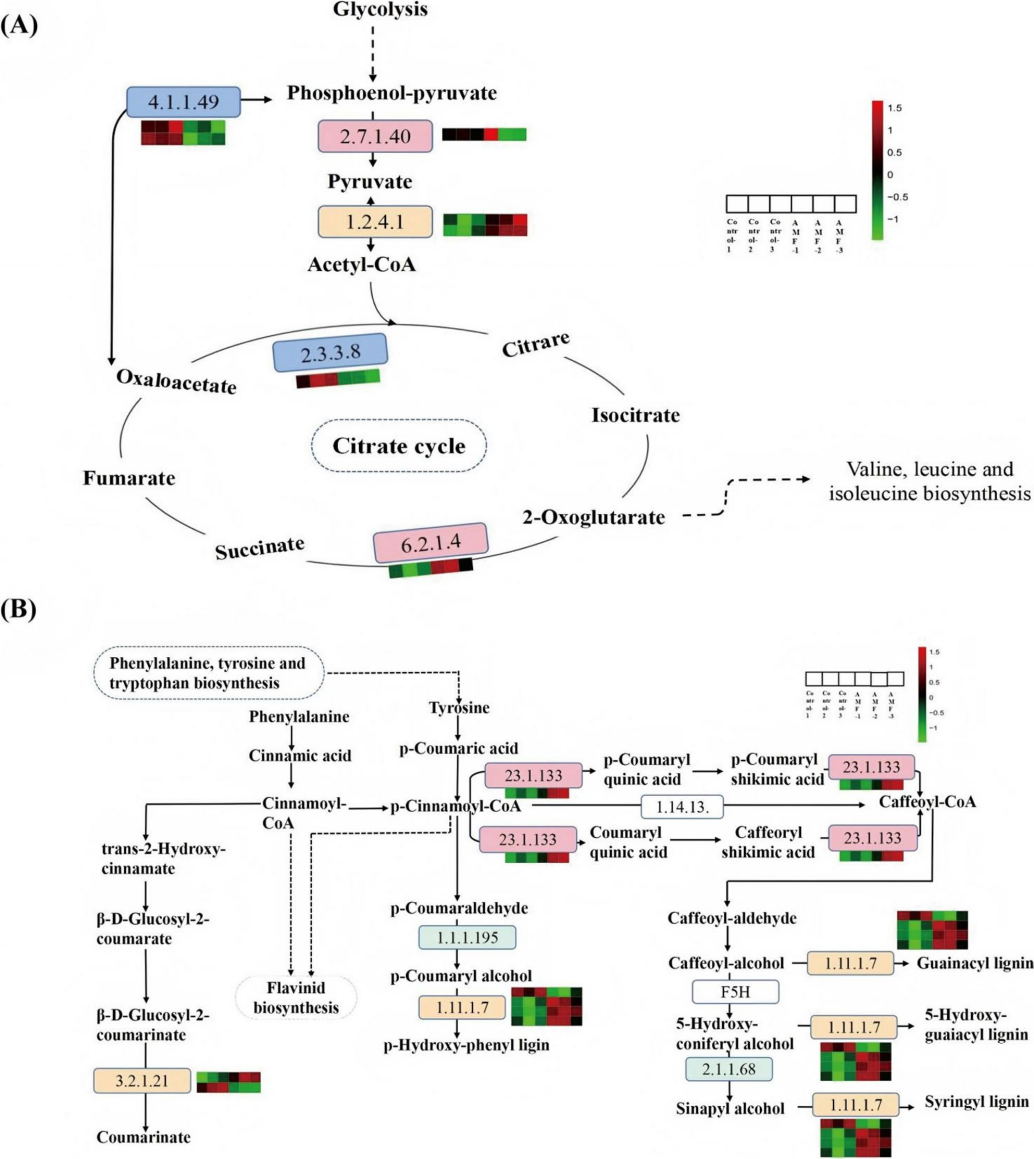
The responses of pyruvate metabolism, the citric acid cycle (TCA) (**A**), and phenylpropanoid biosynthesis pathways (**B**) to AMF inoculation have changed. The proteins identified as showing no significant changes in the pathway are indicated by green rectangles. Blue rectangles denote differentially expressed downregulated proteins, while pink rectangles denote differentially expressed upregulated proteins. Yellow rectangles indicate functional components in the pathway for which multiple proteins include both upregulated and downregulated differentially expressed proteins. The red and green squares beside each protein represent its differential expression in each sample, with red indicating upregulation and green indicating downregulation

**Fig. 5:**
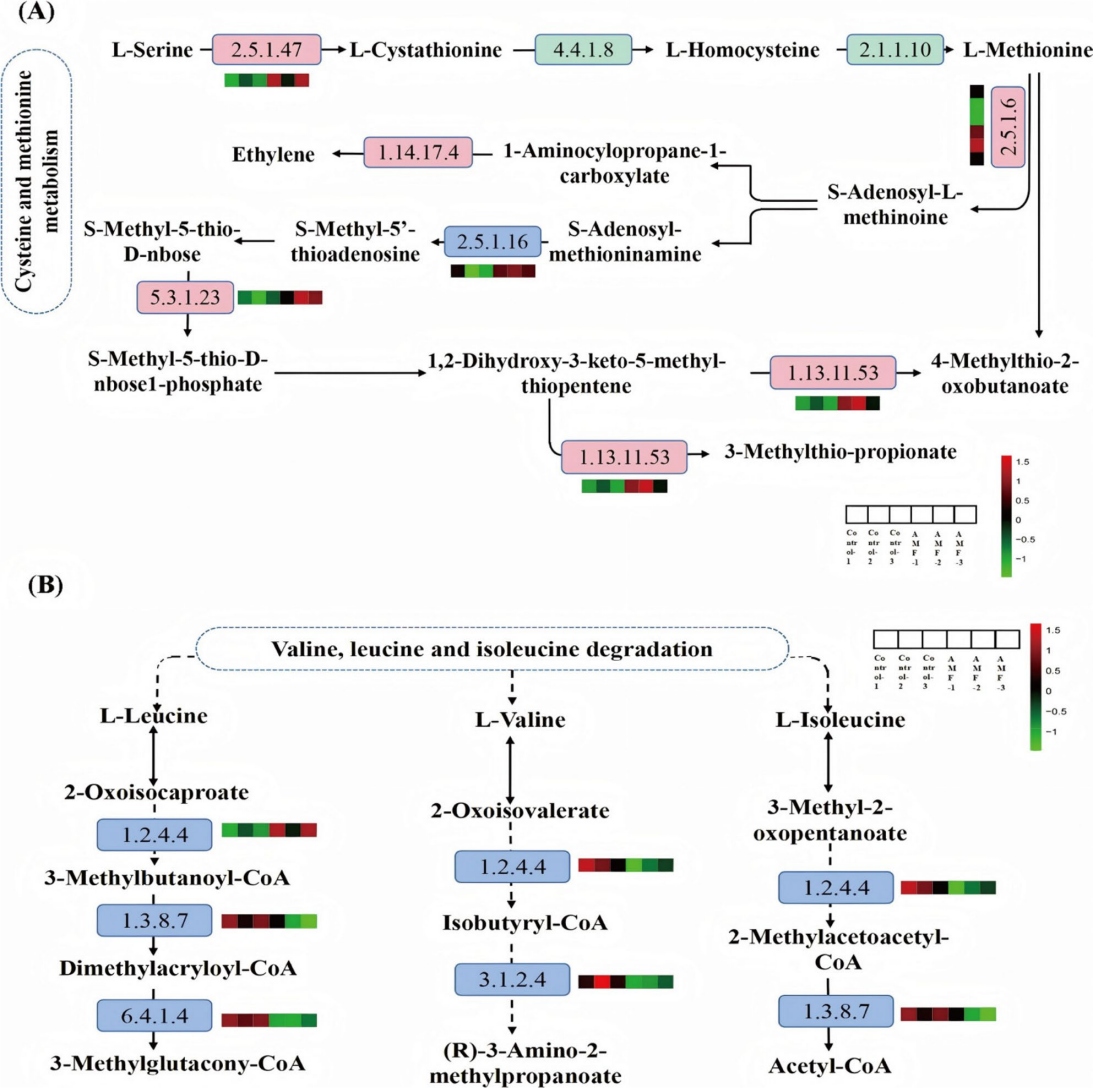
Changes in amino acid metabolic pathways in response to AMF inoculation. **A** Changes in the metabolic pathways of cysteine and methionine. **B** Changes in the degradation pathways of valine, leucine, and isoleucine. The proteins identified as showing no significant changes in the pathway are indicated by green rectangles. Blue rectangles denote differentially expressed downregulated proteins, while pink rectangles denote differentially expressed upregulated proteins. The red and green squares beside each protein represent its differential expression in each sample, with red indicating upregulation and green indicating downregulation

Based on the KEGG enrichment analysis results, we further examined the DEPs involved in the AMF-induced metabolic processes in *P. quinquefolius*. Compared to the control group, significant changes were observed in metabolic pathways such as pyruvate metabolism, the citric acid cycle (TCA), phenylpropanoid biosynthesis, and amino acid pathways in the AMF-inoculated group (Figs. 4 and 5). A total of six DEPs related to pyruvate metabolism and the TCA cycle were identified (Fig. 4A), including one pyruvate kinase (PK, EC: 2.7.1.40; Prot_1629), three components of pyruvate dehydrogenase E1 (aceE, EC: 1.2.4.1; Prot_467, Prot_43152, and Prot_73), and two phosphoenolpyruvate carboxykinases (ATP) (pckA, EC: 4.1.1.49; Prot_45092, Prot_20832). Additionally, two differentially expressed proteins involved in the citric acid cycle were identified: ATP citrate (pro-S) lyase (EC: 2.3.3.8, ACLY) and succinyl-CoA synthetase alpha subunit (EC: 6.2.1.4, sucD). The results indicate that proteins related to pyruvate metabolism and the TCA cycle are regulated following AMF inoculation.

In addition, seven DEPs involved in phenylpropanoid biosynthesis were identified (Fig. 4B). Among these, two are related to β-glucosidase (EC: 3.2.1.21; Prot_20859, Prot_12151), which are upstream enzymes in coumarin biosynthesis and showed upregulated expression following AMF inoculation. Furthermore, following AMF inoculation, the number of proteins involved in lignin biosynthesis significantly increased, including four types of peroxidases (EC: 1.11.1.7; Prot_13280, Prot_36790, Prot_3881, Prot_22582). These findings indicate that AMF inoculation induces the expression of proteins related to the biosynthesis of coumarins and lignin.

It is noteworthy that significant changes also occurred in the amino acid metabolism pathways (Fig. 5). The results showed that AMF inoculation upregulated the expression of several amino acid synthesis-related enzymes, including cysteine synthase (EC:2.5.1.47, cysK), S-adenosylmethionine synthetase (EC:2.5.1.6, metK), and methylthioribose-1-phosphate isomerase (EC:5.3.1.23, mtnA) (Fig. 5A). As a central hub between primary and secondary metabolism, the synthesis pathways of cysteine and methionine converted carbon flow into sulfur-containing amino acids, thereby providing essential sulfur donors for protein synthesis and the generation of various metabolic products. AMF inoculation downregulated the expression of 2-oxoisovalerate dehydrogenase E1 component alpha subunit (EC: 1.2.4.4), acyl-CoA dehydrogenase (EC: 1.3.8.7), 3-methylbutyryl-CoA carboxylase alpha subunit, and 3-hydroxyisobutyryl-CoA hydrolase (EC: 3.1.2.4), thereby slowing the degradation of valine, leucine, and isoleucine (Fig. 5B). In summary, these results indicate that *P. quinquefolius* responds to AMF inoculation, with proteins involved in various metabolic pathways being regulated by AMF, leading to significant changes in their expression levels and enriched pathways.

### Differential protein interaction networks and domain analysis

**Fig. 6:**
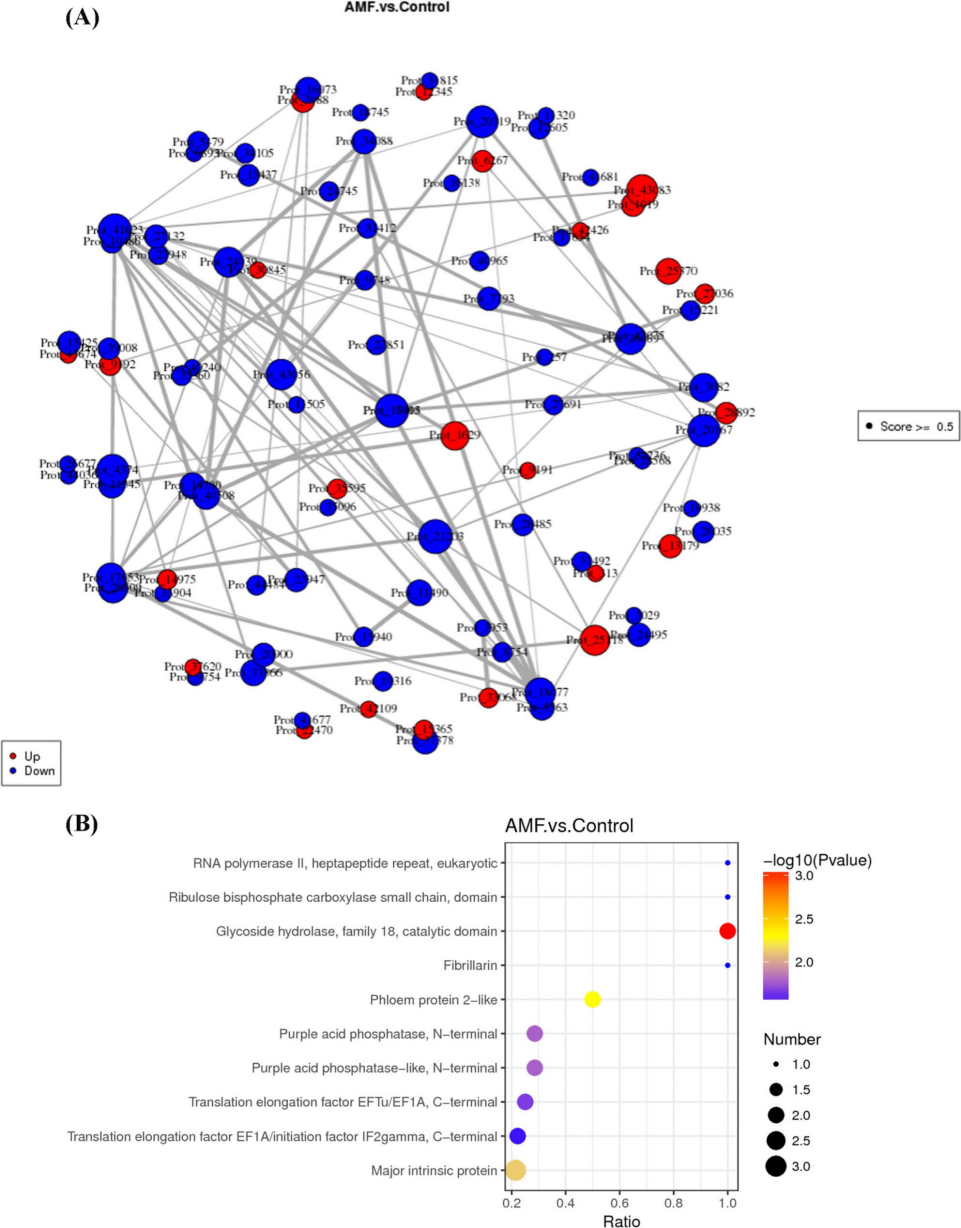
Protein-protein interactions and domain analysis. **A** Protein-protein interaction network of differentially expressed proteins (DEPs). In the interaction network, each node represents a protein, and the size of the node indicates the number of interacting proteins; a larger node signifies a higher number of interacting proteins. The color of the node represents the expression level of the protein in comparison groups, where red indicates that this protein is significantly highly expressed in the AMF treatment group and green indicates that this protein is significantly low expressed. A score value of score > 0.5 indicates a higher confidence in the protein interaction; **B** Bubble plot of the structural domains of differentially expressed proteins

To clearly illustrate the protein-protein interactions among the DEPs, a protein interaction network was constructed using the proteins with higher abundance (Fig. 6A). Among the upregulated proteins, nine distinct proteins interacted with pyruvate kinase Prot_1629; eight proteins interacted with Prot_43083. Additionally, four proteins interacted with Prot_1019 and Prot_43083. In the downregulated proteins, five proteins interacted with Prot_3082 were identified. The results indicated significant interactions between DEPs. These proteins are primarily involved in processes such as cellular material metabolism, binding, signal transduction, and protein translation, with most functioning as enzymes and structural proteins.

Protein domains are repetitive units within protein molecules that reflect the evolutionary history of proteins. To assess whether the protein families changed after AMF inoculation, we analyzed the domains present in the DEPs (Fig. 6B, Table S5). Family 18 glycoside hydrolases, catalytic domains, carotenoid dioxygenases, translation elongation factor EFTu/EF1A C-terminal domains, translation elongation factor EF1A/initiation factors, and the small chain domain of ribulose bisphosphate carboxylase were enriched after AMF inoculation. The results indicate that the DEPs affected by AMF treatment exhibit functional modularization characteristics within the interaction network, primarily enriched in biological processes such as gene transcription, protein translation, and plant metabolism.

### Association analysis of differential proteins and genes in AMF regulation of *P. quinquefolius* metabolic pathways

**Fig. 7:**
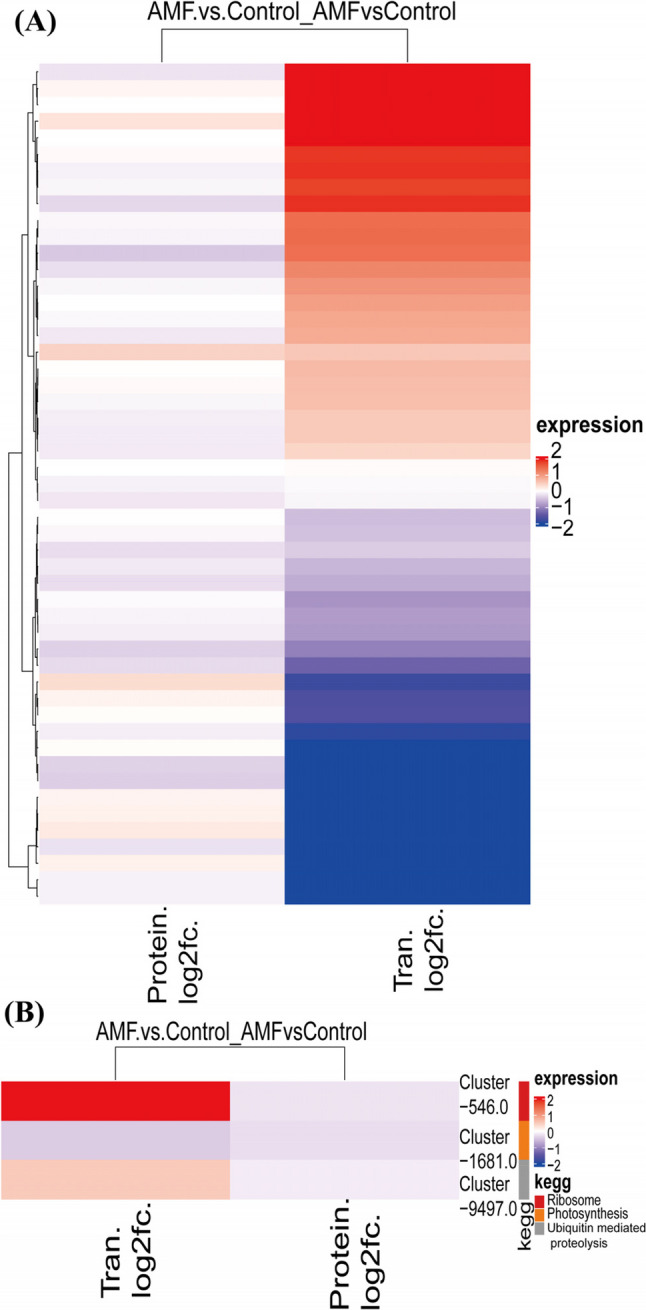
Differential protein and differential gene association analysis. **A** Clustering analysis of the expression patterns of the associated differential protein group and transcriptome; **B** KEGG pathway enrichment diagram of differentially expressed genes and differential proteins

Compared to the non-inoculated group, a total of 124 upregulated genes and 461 downregulated genes were identified in the AMF-inoculated group (Figure S2). Based on sequence homology, the DEGs were annotated and categorized into three Gene Ontology (GO) categories (Figure S3). To reveal the relationships between the transcriptome and proteome in the regulation of *P. quinquefolius* primary metabolism by AMF, we conducted an integrated analysis of differentially expressed genes and proteins. We identified 51 associated differentially expressed genes and proteins and performed a clustering analysis (Fig. 7A). We found 6 genes that were significantly upregulated at both the transcript and protein levels, and 16 genes that were downregulated at both levels, indicating a positive correlation between omics (Table S6). A KEGG pathway enrichment analysis was conducted for the DEGs/DEPs (Fig. 7B), revealing enrichments in the pathways of Photosynthesis (map00195), Ribosome (map03010), and Ubiquitin-mediated proteolysis (map04120). Among them, the differential genes and proteins in the Photosynthesis pathway (Cluster-9497.0/Prot_51078), Ribosome pathway (Cluster-546.0/Prot_49976), and Ubiquitin-mediated proteolysis pathway (Cluster-1681.0/Prot_26531) play a role in the process by which AMF affects *P. quinquefolius*’s primary metabolism. These results provide important clues for understanding the molecular mechanisms by which AMF regulates *P. quinquefolius* metabolism, suggesting that the synergy and differences in gene expression and protein accumulation jointly participate in plant responses to AMF.

### Association analysis of differential proteins and metabolites in AMF regulation of *P. quinquefolius* metabolic pathways

**Fig. 8:**
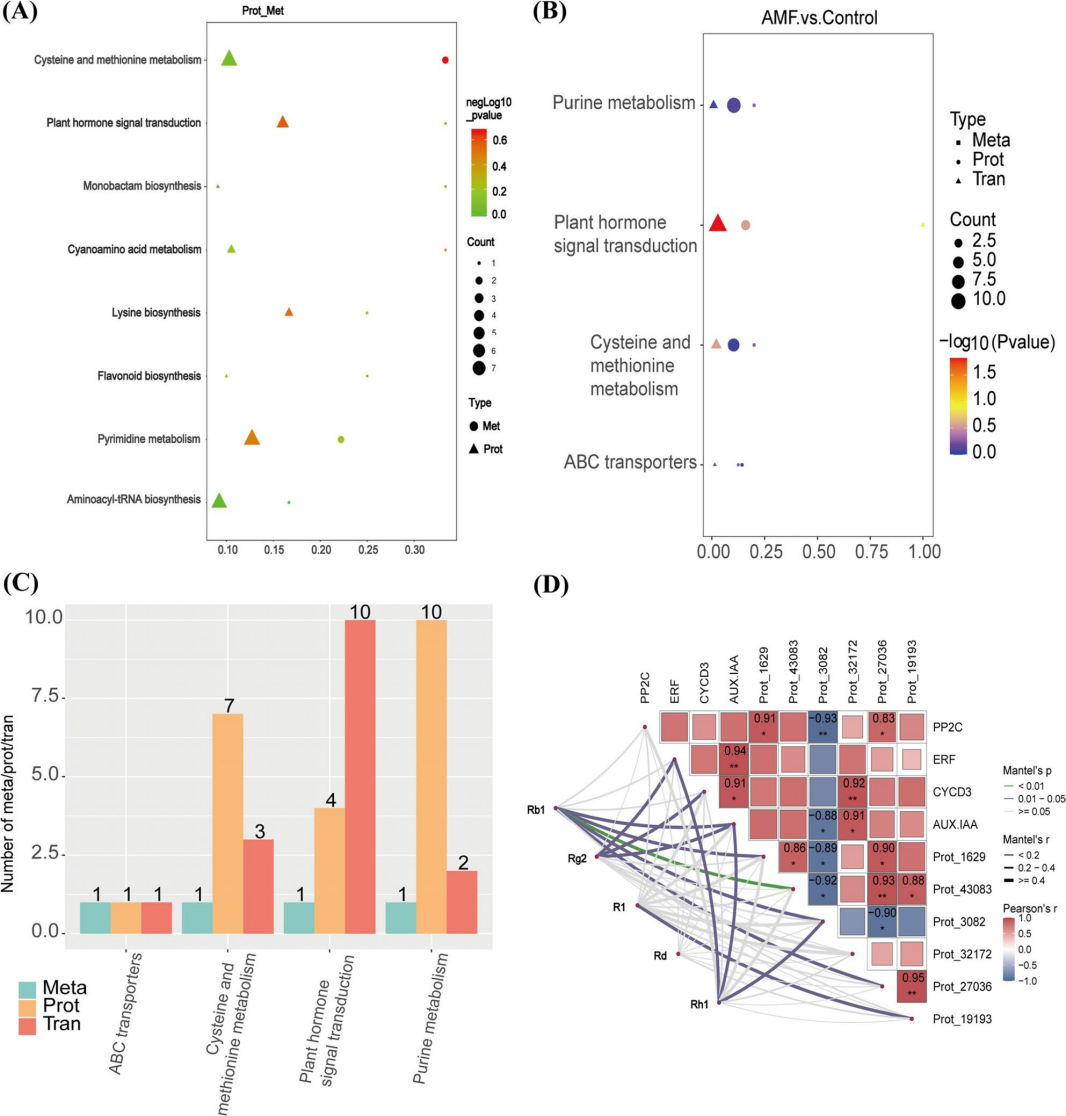
Association analysis of differential proteins (Prot), differential genes (Tran), and differential metabolites (Meta). **A** KEGG pathway enrichment diagram for differentially expressed proteins and differential metabolites; **B** KEGG pathway enrichment diagram for differential proteins, differential genes, and differential metabolites; **C** A graphical representation of the enrichment counts of differential genes, differential proteins, and differential metabolites across different pathways; **D** The mantel-test correlation between DEPs, DEGs, and DEMs. The left side displays the ginsenoside components. The colors of the lines connecting the triangles represent different meanings: blue indicates significant correlation (*P* < 0.05); green indicates highly significant correlation (*P* < 0.01); gray indicates no correlation (*P* > 0.05). In the color of the boxes, red represents positive correlation, while blue represents negative correlation

The top 50 differentially expressed proteins (DEPs) and top 50 DEMs were selected for correlation analysis (Figure S4). The results show a significant correlation between differential proteins and differential metabolites. To identify the biological pathways involved in AMF regulation of *P. quinquefolius* secondary metabolism, KEGG pathway enrichment analysis was conducted on the DEMs/DEPs (Fig. 8A). It was found that pathways such as cysteine and methionine metabolism, Plant hormone signal transduction, and Lysine biosynthesis were significantly enriched. Specifically, there were 2 differential metabolites related to cysteine and methionine metabolism: glutathione and L-aspartic acid, along with 5 differential proteins (cysteine synthase (Prot_32172), S-adenosylmethionine synthase (Prot_27036), aminocyclopropane carboxylic acid oxidase (Prot_19193), methylthio-1-phosphate isomerase (Prot_9204), and 1,2-dihydroxy-3-keto-5-methylthio-2-pentene dioxygenase (Prot_31841)). In this pathway, AMF inoculation induced the upregulation of cysteine synthase, which correspondingly resulted in an increase in glutathione levels detected by metabolomics analysis (Table S7). The differential metabolite associated with the plant hormone signal transduction pathway was jasmonic acid (JA), along with 4 differential proteins (ethylene receptor (Prot_28436), ethylene-insensitive protein 2 (Prot_42075, Prot_24035), and the regulatory protein NPR1 (Prot_8923)). This indicates that AMF can regulate the corresponding metabolic pathways by upregulating proteins such as cysteine synthase and ethylene receptors, thereby increasing the levels of metabolites such as glutathione and jasmonic acid.

### Association analysis of differential proteins, genes, and metabolites in AMF regulation of *P. quinquefolius *metabolic pathways

The results showed that the KEGG combined analysis was primarily enriched in four core pathways: ABC transporters, Cysteine and methionine metabolism, Plant hormone signal transduction, and Purine metabolism (Fig. 8B). It also indicated the number of enriched differential metabolites, differential genes, and differential proteins in each pathway (Fig. 8C). The results revealed that in the cysteine and methionine metabolism pathway, differential genes and proteins involved in the synthesis of cysteine synthase (EC:2.5.1.47/Prot_32172), S-adenosylmethionine synthetase (EC:2.5.1.6/Prot_27036) and aminocyclopropanecarboxylate oxidase (ACC oxidase) (EC:1.14.17.4/Prot_19193) were significantly upregulated (Figure S5). In addition, the expression of genes PP2C (Cluster-433.30485), ERF1/2 (Cluster-433.2059), and CYCD3 (Cluster-433.39460) in the plant signaling transduction pathways is significantly upregulated (Figure S6).

**Fig. 9:**
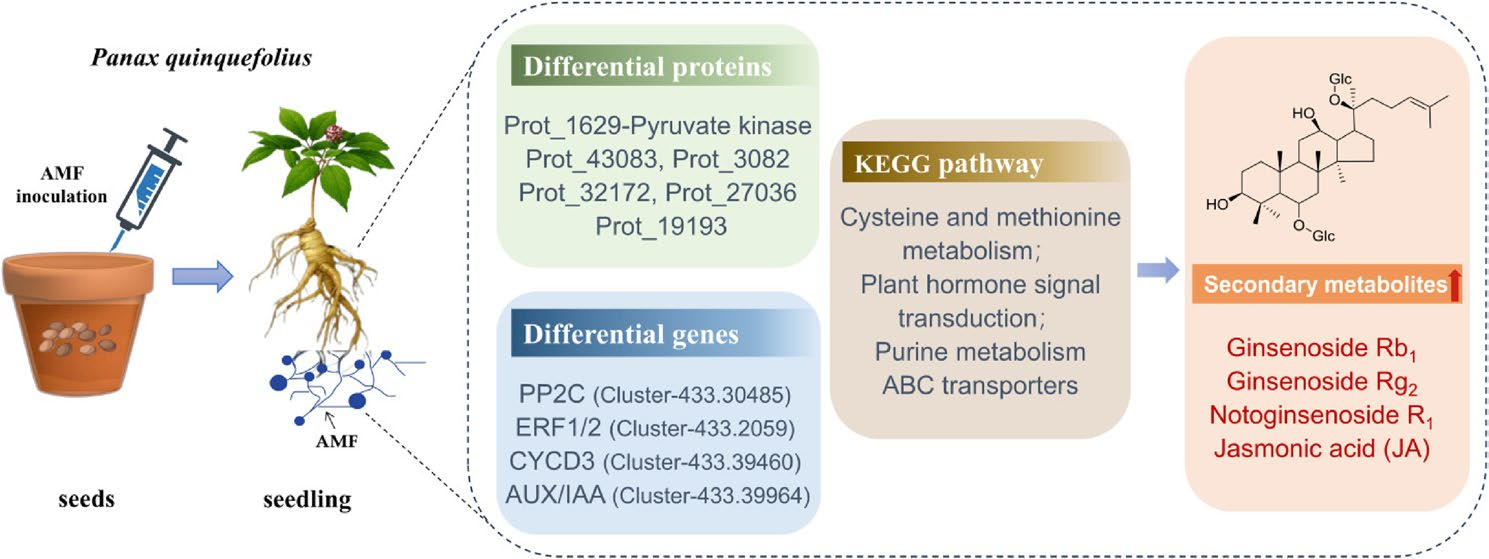
Schematic diagram of the mechanism by which AMF affects the metabolic products of *P. quinquefolius*. AMF inoculation alters proteins and genes, affecting metabolic pathways, which in turn influences the accumulation of secondary metabolites in *P. quinquefolius*

A correlation analysis was conducted among the three DEPs with the most protein network interaction connections (Prot_1629, Prot_43083, Prot_3082), the significantly upregulated DEPs in the co-enriched KEGG pathways (Prot_32172, Prot_27036, Prot_19193), the upregulated DEGs (PP2C, ERF, CYCD3, AUX/IAA), and the secondary metabolites in the DEMs, specifically the ginsenosides (Fig. 8D). The results revealed that Ginsenoside Rb_1_, which ranks highly in terms of differences, has a significant positive correlation with AUX/IAA, Prot_1629, Prot_43083, Prot_27036, and Prot_19193, and a significant negative correlation with Prot_3082. Rg_2_ shows a significant positive correlation with ERF, CYCD3, and AUX/IAA. Rh_1_ is significantly positively correlated with ERF and AUX/IAA, while it is significantly negatively correlated with Prot_3082. R_1_ demonstrates a significant positive correlation with Prot_19193. These results indicated that AMF inoculation regulated the expression of DEPs and DEGs, which are key substances in the secondary metabolism synthesis pathway and had a relevant impact on the accumulation of ginsenosides (Fig. 9).

## Discussion

The results of this study indicated that inoculation with AMF promoted the growth of *P. quinquefolius* and significantly affected its protein expression. The differentially expressed proteins identified were primarily located in the cytoplasm and nucleus, such as β-glucosidase and peroxidase. GO and KEGG functional enrichment analyses revealed that the DEPs possessed various molecular functions and were involved in multiple biochemical metabolic pathways regulated by AMF, mainly including the biosynthesis of nitrogen metabolism, carbon fixation in photosynthetic organisms, phenylpropanoid biosynthesis, and pyruvate metabolism. Multi-omics integrative analysis suggested that S-adenosylmethionine synthetase and ACC oxidase in the cysteine and methionine metabolism pathways played crucial roles in the regulation of secondary metabolism in *P. quinquefolius* by AMF.

This study screened and identified DEPs that underwent significant changes after AMF inoculation. The volcano plot highlighted the top 9 significantly different proteins. Among the upregulated DEPs, Prot_28892 belongs to translationally controlled tumor protein (TCTP), which enhances the ability to scavenge reactive oxygen species (ROS) and regulates hormonal signaling pathways to bolster plant resistance [38]. Prot_3881 is haem peroxidase, which plays a crucial role in plant growth and development as well as in stress adaptation by scavenging hydrogen peroxide (H_2_O_2_) and participating in secondary metabolism pathways [39]. Research indicated that the key enzyme S-adenosylmethionine synthetase, identified in the results of multi-omics analysis, could provide methyl donors for plants, driving the synthesis of various secondary metabolites [40]. ACC oxidase was shown to differentially regulate the accumulation of metabolites such as flavonoids through ethylene signaling [41]. Additionally, GO functional analysis indicated significant enrichment of peroxidase activity proteins, oxidoreductase proteins and transport proteins, while KEGG analysis showed significant enrichment of phenylpropanoid biosynthesis pathways (Fig. 3). These results indicate that AMF inoculation regulates the synthesis pathways of secondary metabolites in *P. quinquefolius* and the expression of key proteases involved in these pathways.

This study indicates that AMF significantly affects the expression of proteins involved in pyruvate metabolism and the TCA cycle. As core pathways of plant energy metabolism, pyruvate metabolism and the TCA cycle are not only integral to energy supply but are also closely related to metabolic pathways such as photosynthesis, fatty acid synthesis, and amino acid metabolism, and are universally present in various organs and tissues of plants [42, 43]. In the pyruvate metabolism pathway, pyruvate kinase (PK), a key regulatory enzyme in this pathway, irreversibly converts adenosine diphosphate (ADP) and phosphoenolpyruvate (PEP) into adenosine triphosphate (ATP) and pyruvate, with its protein abundance tightly correlated with pyruvate levels [44]. Pyruvate produced through PK catalysis participates in various metabolic pathways within the plant and acts as a critical precursor in cellular metabolic processes [45]. The results of this study showed that, compared to the control, the expression level of PK significantly increased after AMF inoculation. Notably, pyruvate kinase Prot_1629 plays a prominent role in the differential protein interaction network. These findings suggest that AMF inoculation enhances the activity of pyruvate kinase, providing a basis for the biosynthesis of pyruvate and downstream secondary metabolites in *P. quinquefolius*. Additionally, pyruvate enters the mitochondria and forms acetyl-CoA under the action of pyruvate dehydrogenase, subsequently entering the TCA cycle. The TCA cycle is an important pathway for aerobic respiration in plants and a primary source of raw materials for photosynthesis, mainly converting pyruvate to malate to produce ATP, which is significant for maintaining the energy balance and material metabolism in plants [46]. Research by Gupta et al. demonstrated that AMF colonization significantly increased the activity of crucial enzymes such as succinate dehydrogenase, fumarate dehydrogenase, and malate dehydrogenase, which participate in the TCA cycle in arsenic-affected wheat [47]. Kchikich et al. [48] reported that AMF induced changes in carbon and nitrogen metabolism pathways, significantly increasing the activity of key enzymes such as glutamate dehydrogenase and aconitase in sorghum, which are involved in the TCA cycle. This study found that the DEPs involved in the TCA pathway were significantly regulated, and these DEPs, along with DEGs, were significantly enriched in the photosynthetic pathway. This indicates that AMF inoculation enhances the expression of proteins and genes related to plant photosynthesis. This aligns with our previously reported findings that AMF inoculation can significantly increase the net photosynthetic rate and the content of chlorophyll, Rubisco, and soluble carbohydrates in *P. quinquefolius* [28].

This study indicates that AMF significantly affects the expression of proteins involved in phenylalanine metabolism. Phenylalanine metabolism is one of the important secondary metabolic pathways in plants, playing a crucial role in plant growth and development as well as in plant-environment interactions [49, 50]. Research by Najar et al. found that AMF increased the activity of phenylalanine ammonia-lyase (PAL) in spinach, playing a key role in the phenylalanine metabolic pathway and the synthesis of secondary metabolites [51]. The production of lignin is an important branch of the phenylalanine metabolic pathway and is a major component of plant cell walls, participating in various biological processes such as the formation of water and mineral-conducting vessels, resistance to pathogen invasion, and tolerance to abiotic stress. These processes have a significant impact on plant growth, development, and adaptation to environmental conditions [52–55]. Studies have shown that lignin synthesis involves the participation of various key enzymes, and the expression of critical enzymes such as 4-coumarate: CoA ligase (4CL), hydroxycinnamate: CoA transferase (HCT), cinnamate-CoA reductase (CCR), cinnamyl alcohol dehydrogenase (CAD), and peroxidase (POD) directly or indirectly affects lignin content [56–58]. The results of this study showed that the expression levels of HCT and POD were significantly upregulated after AMF inoculation, and there was an increase in the biomass of *P. quinquefolius*. This indicates that AMF enhances the expression of key enzymes involved in lignin synthesis, providing a basis for promoting plant growth and development.

This study indicates that AMF significantly affects the expression of proteins involved in amino acid metabolism. Amino acids, as precursors for protein synthesis, provide essential nitrogen sources and carbon backbones for plants, participating in the synthesis of biomacromolecules such as chlorophyll and enzymes [59, 60]. The synthesis and metabolism of amino acids are crucial physiological and biochemical processes during plant growth and development, with cysteine and methionine metabolism being important branches of this pathway [61–63]. In plants, the synthesis of cysteine begins from intermediates of glycolysis and is transformed into methionine through cysteine and methionine metabolism; methionine acts as a precursor for hormones that promote plant metabolism [64]. Research by Torres et al. revealed that mycorrhizal symbiosis alters the metabolite content in grapes and that AMF inoculation significantly increases the levels of glucose and amino acids in grapes [65]. Cun et al. clarified that AMF can improve the soil environment of *Glycine max* L., enhance soil enzyme activity, and promote plant growth [66]. Wu et al. found that AMF inoculation can enhance nitrogen uptake and assimilation, promoting the synthesis and accumulation of various amino acids, thereby improving the resistance of tea plants to drought stress [67]. In this study, DEPs related to cysteine synthase (EC:1.14.17.4) and S-adenosylmethionine synthase (metK) in the amino acid metabolism pathway were significantly upregulated after AMF inoculation. Furthermore, multi-omics analysis revealed that proteins and genes involved in encoding S-adenosylmethionine synthetase and ACC oxidase were also significantly upregulated. In contrast, the expression of proteins involved in the degradation pathways of valine, leucine, and isoleucine was significantly downregulated, which slowed down amino acid degradation. These results suggest that AMF enhances the expression of key enzymes involved in amino acid synthesis and maintains the stability of amino acid content, providing precursor materials for protein synthesis during plant growth and metabolic processes.

Although this study clarified the key nodes and pathways through which AMF regulates the secondary metabolism of *P. quinquefolius* at the protein level, certain limitations remained. Firstly, the experiments were conducted under controlled greenhouse conditions, which do not fully mimic the complex interactions of soil microbial communities and climatic factors in the field environment. Future research should carry out multi-year, multi-site field trials to verify the generalizability of the conclusions. Secondly, this study selected only a single AMF species, *Rhizophagus intraradices*, while the diversity of AMF in nature is extensive, and different species may exhibit varying regulatory effects on host metabolism. Subsequent studies should compare the synergistic or specific effects of multiple AMF species. Furthermore, the experimental period was 12 weeks, covering only the growth stage of *P. quinquefolius* seedlings and failing to reveal the dynamic regulatory patterns of AMF throughout the entire growth period and the metabolism of perennial root systems. Future research should combine long-term positioning trials with multi-omics analyses to further dissect the secondary metabolism regulatory network mediated by AMF and its ecological adaptability mechanisms.

## Conclusion

After treatment with arbuscular mycorrhizal fungi (AMF), the growth and protein profile of *P. quinquefolius* underwent significant changes. Key metabolic pathways involving pyruvate metabolism, phenylpropanoid biosynthesis, and amino acid metabolism were notably regulated by AMF inoculation. Multi-omics analysis revealed that proteins such as S-adenosylmethionine synthetase, which is involved in cysteine and methionine metabolism, and genes like CYCD3, involved in plant hormone signal transduction, were significantly upregulated, with their expression levels showing a significant positive correlation with ginsenoside accumulation. This study, through integrated proteomics and multi-omics analysis, elucidated the critical nodes and pathways of AMF regulation in the secondary metabolism of *P. quinquefolius*, provided new insights into the regulatory network of AMF interactions with medicinal plants, and offered data support for the expansion of AMF applications in the cultivation of traditional Chinese medicinal materials.

## Supplementary Information

Supplementary Material 1.

## Data Availability

The data that support the findings of this study are available from the corresponding author upon reasonable request.

## Abbreviations

AMF: Arbuscular mycorrhizal fungi
TMT: Tandem mass tag
GO: Gene ontology
KEGG: Kyoto encyclopedia of genes and genomes
DEPs: Differentially expressed proteins
DEGs: Differentially expressed genes
DEMs: Differentially expressed metabolites
PPFD: Photosynthetic photon flux density
LC-MS/MS: Liquid chromatography-tandem mass spectrometry
PPIs: Protein-protein interactions
BP: Biological process
CC: Cellular component
MF: Molecular function
TCA: Tricarboxylic acid
