# Supplementary material for: Analysis of key nodes and metabolic pathways in the protein network of secondary metabolism in Panax quinquefolius L. enhanced by arbuscular mycorrhizal fungi
Source: BMC Plant Biol. 2026 Apr 1;26:820. doi: 10.1186/s12870-026-08629-0 (PMC13154870; doi:10.1186/s12870-026-08629-0)
Supplement: Supplementary file 1 — Supplementary Material 1. [file 12870_2026_8629_MOESM1_ESM.docx]

**Analysis of key nodes and metabolic pathways in the protein network of secondary metabolism in *Panax quinquefolius* L. enhanced by arbuscular mycorrhizal fungi**

Yue Wang^1^, Zhifang Ran^1,2^, Siqi Ma^1^, Yuting Yao^1^, Ziqi Liu^1^, Ruzhen Wang^1^, Peng Zhang^3^, Lanping Guo^4*^, Lei Fang^1*^, Jie Zhou^1**^

*^1^School of Biological Science and Technology, University of Jinan, Jinan 250022, PR China*

*^2^Shandong Academy of Traditional Chinese Medicine, Institute of Chinese Materia Medica Resources, Jinan 250013, PR China*

*^3^Shandong Zhongping Pharmaceutical Co. Ltd, Linyi 273399, PR China*

*^4^State Key Laboratory for Quality Ensurance and Sustainable Use of Dao-di Herbs, Beijng 100700, PR China*

***Corresponding author:** guolanpingabc@126.com (LP. Guo); fleiv@163.com (L. Fang)

****Corresponding author:** zhoujie8761@163.com(J. Zhou)

***Supplementary Material***

**1 Supplementary Figures and Tables**

- 1. **Supplementary Figures**


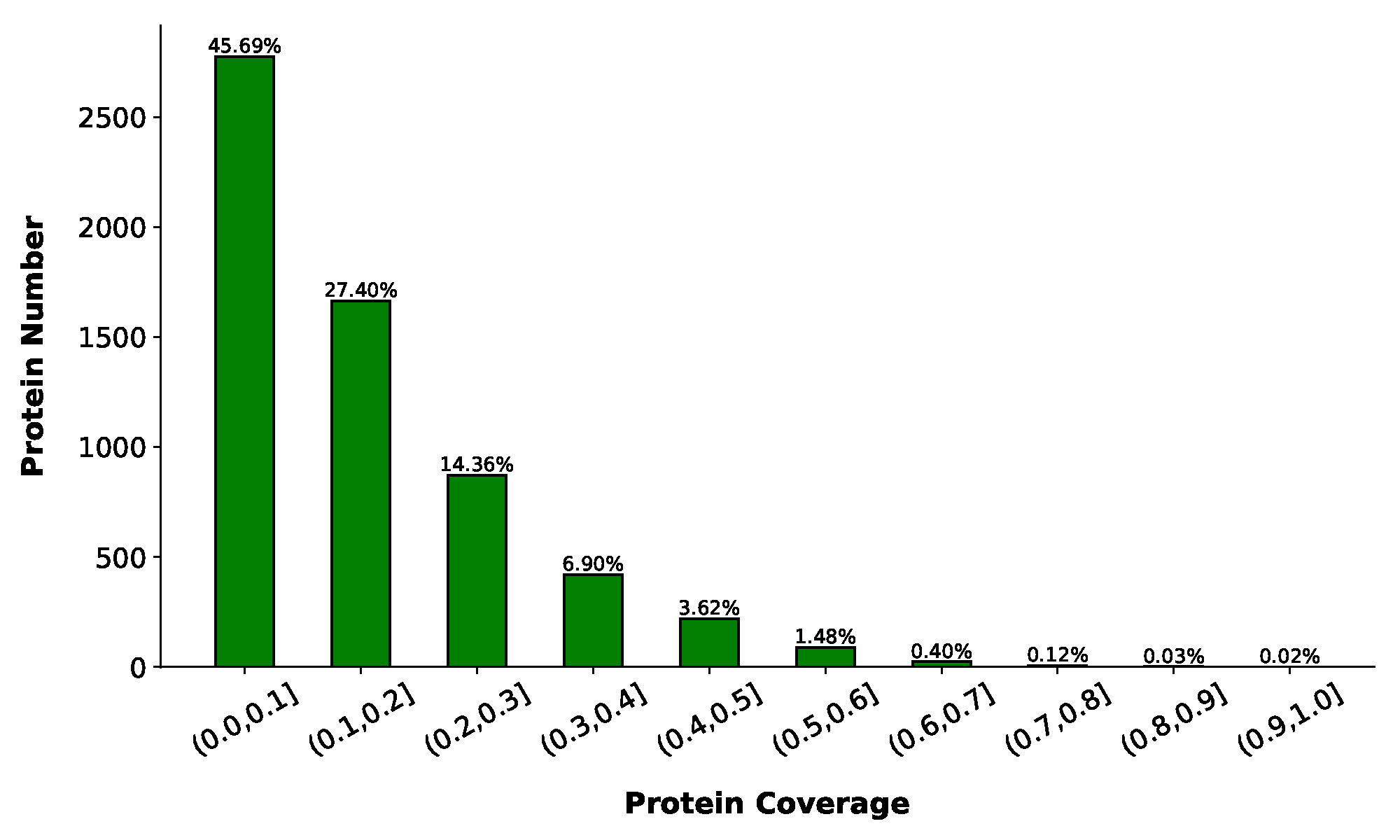

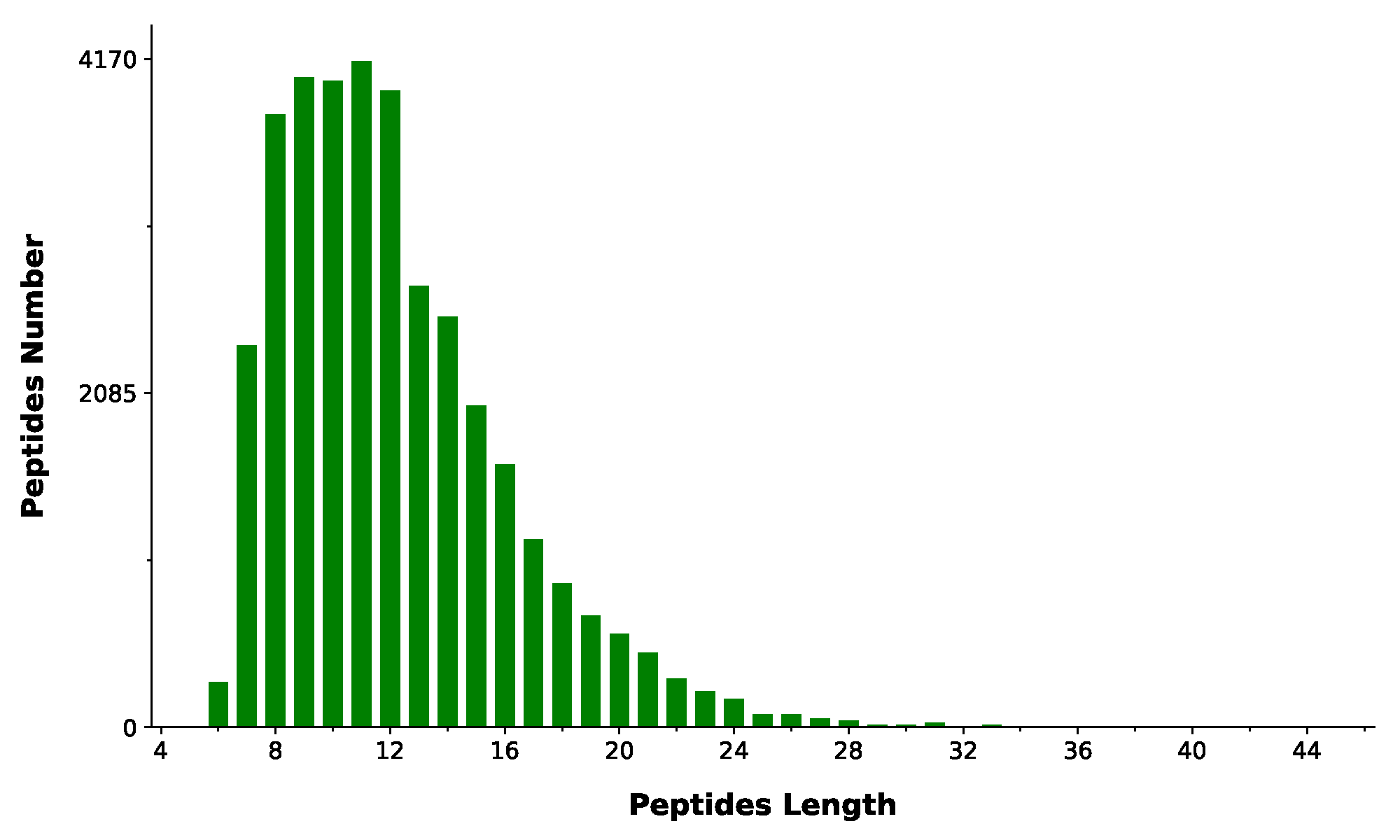


**A**

**B**


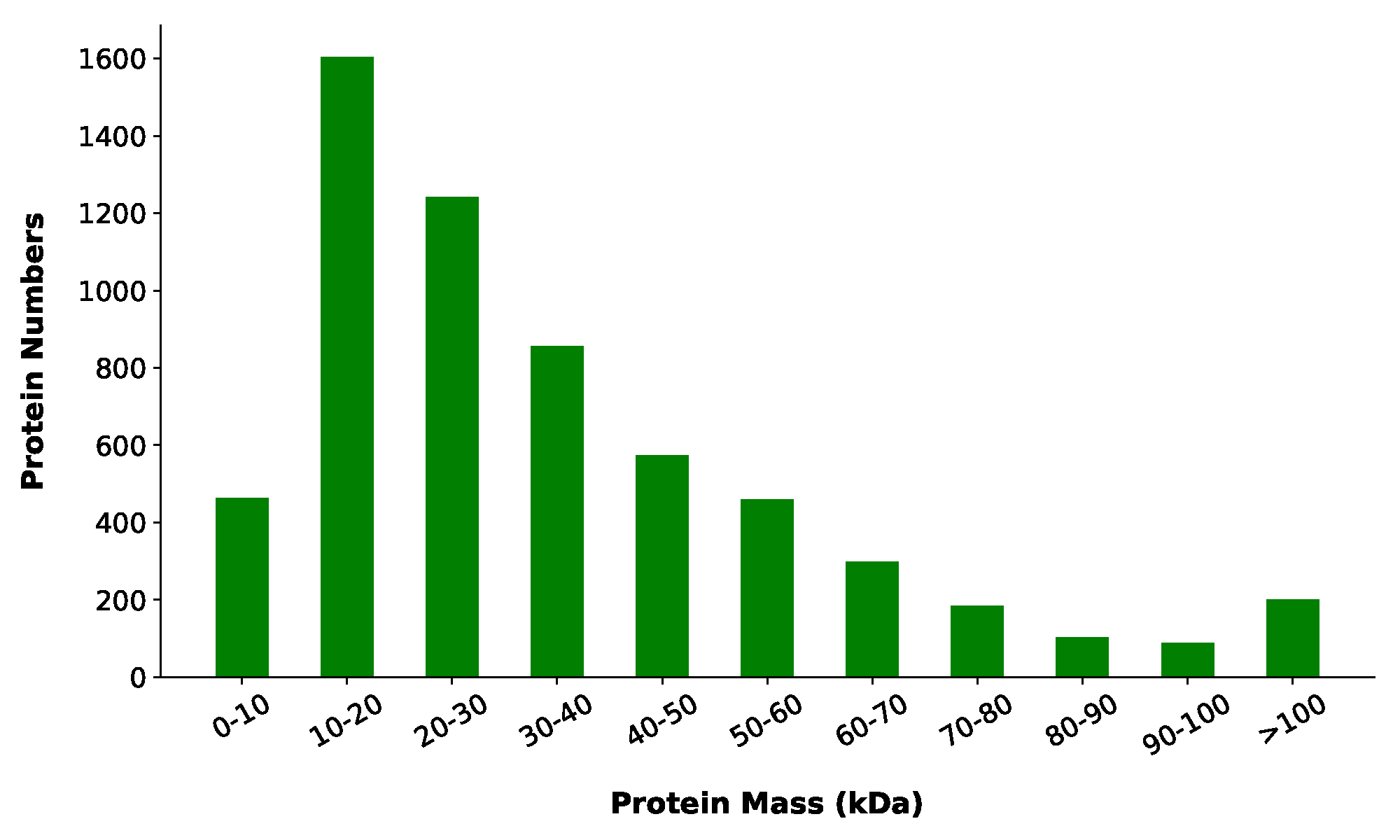


**C**

**Fig. S1** Quality control analysis of TMT sequencing results. (A) Protein sequence coverage distribution. Different colors represent different sequence coverage ranges. (B) Number of peptides per protein distribution. (C) Numbers of unique peptide in proteins


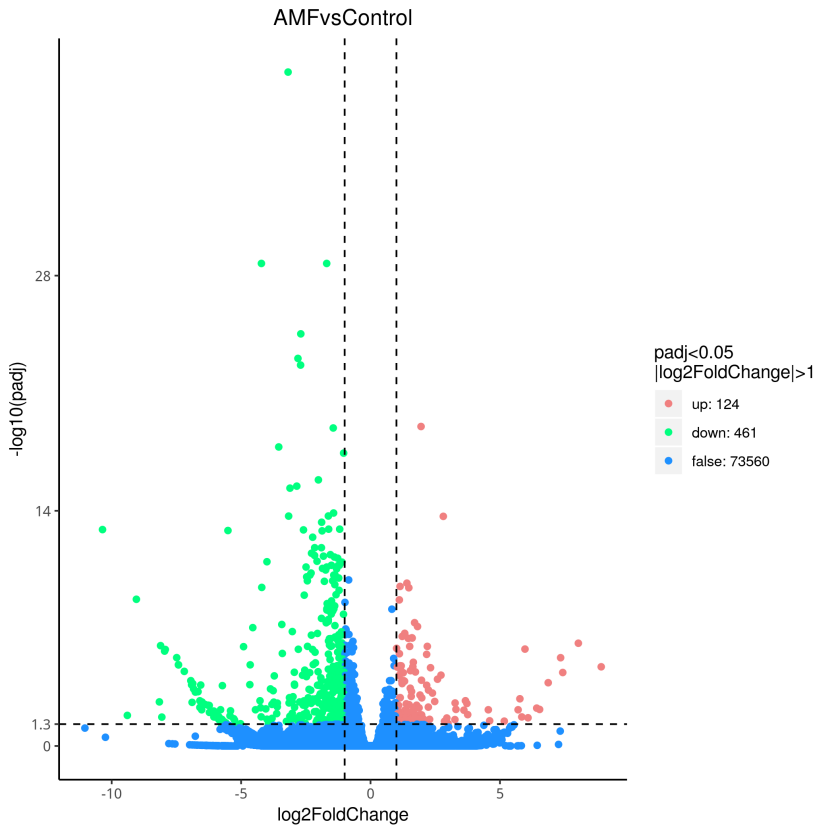


**Fig. S2**  Volcano plot showing the distribution of differentially expressed genes (DEGs) between the AMF group and the control group


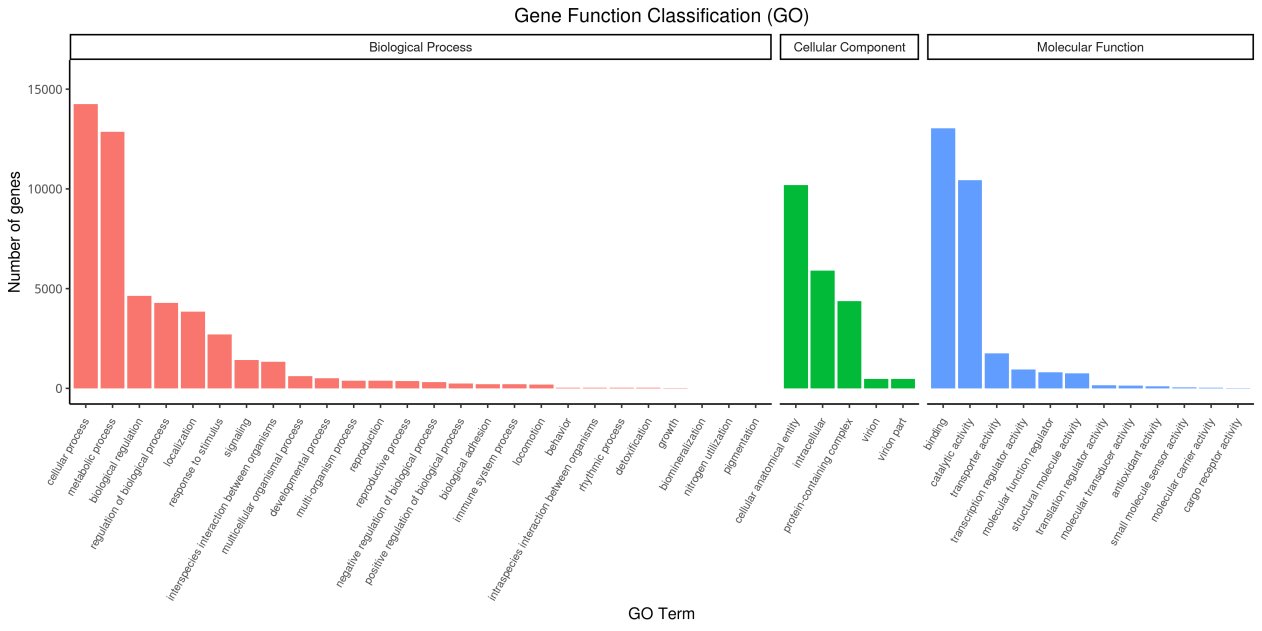


**Fig. S3**  GO enrichment analysis of DEGs


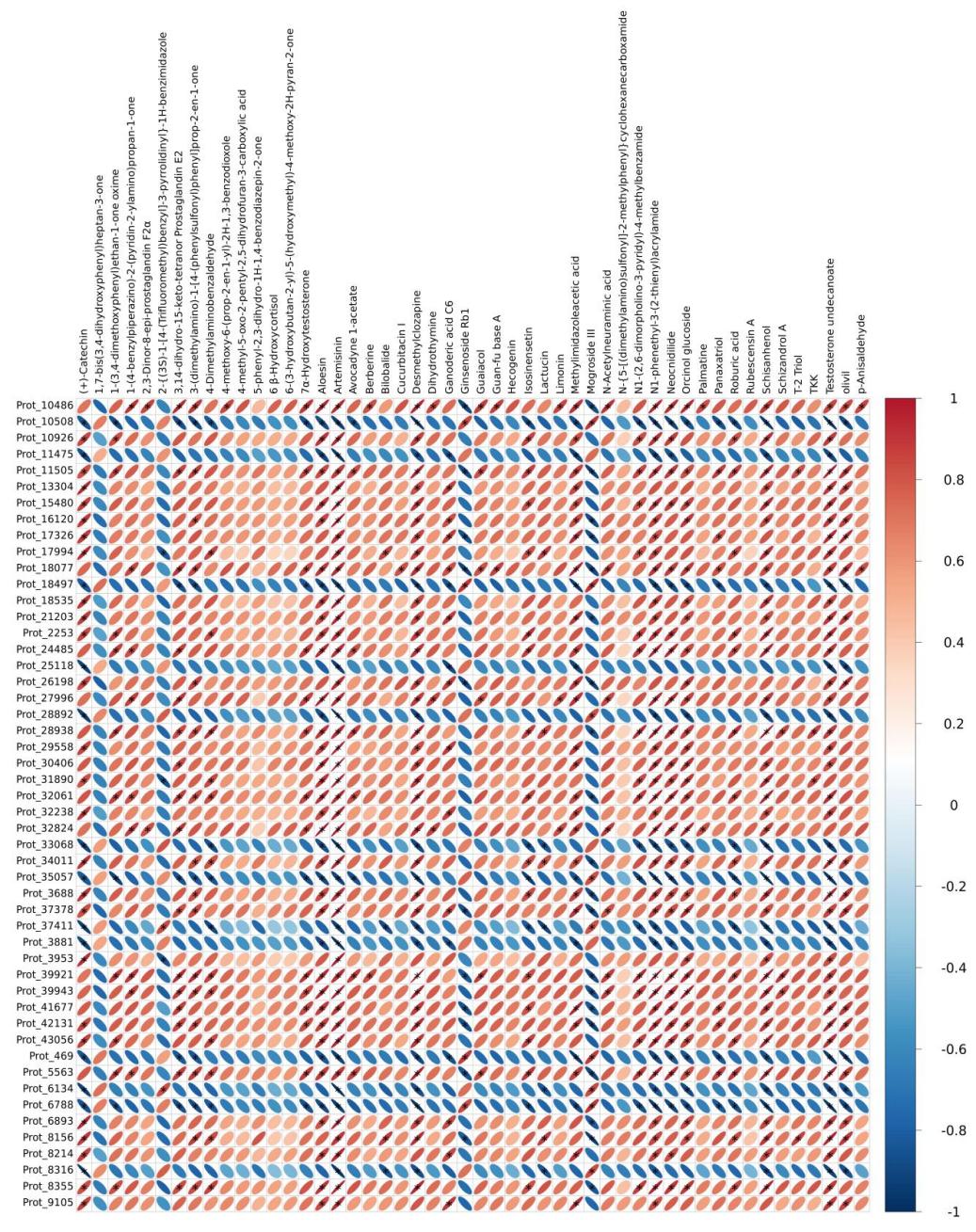


**Fig. S4** Heatmap of Correlation Analysis between Top 50 Differential Proteins and Top 50 Differential Metabolites


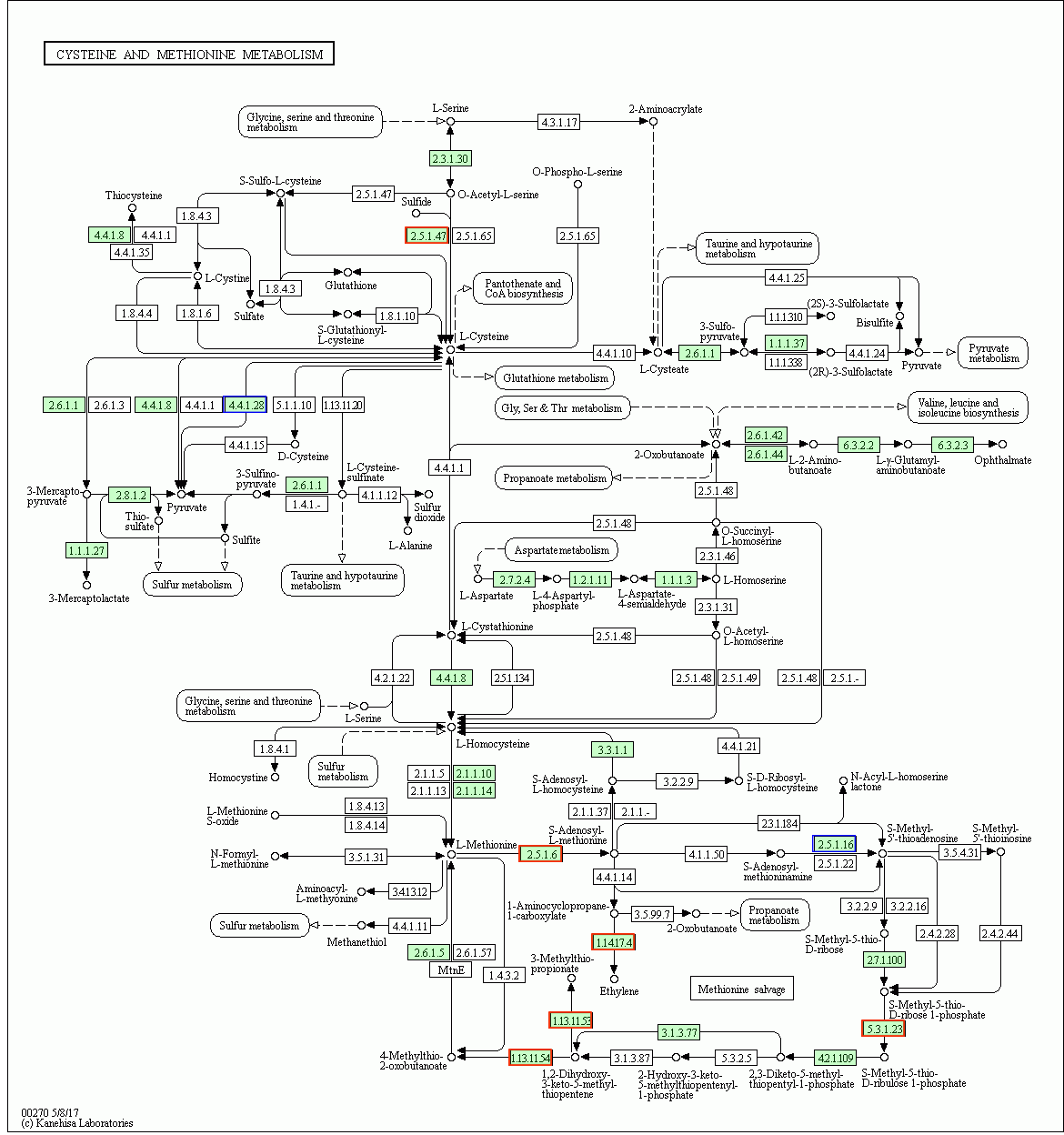


**Fig. S5**  Changes in cysteine and methionine pathways after AMF inoculation. Green represents the enriched DEPs/DEGs. Red boxes indicate significantly upregulated DEPs/DEGs; blue boxes indicate significantly downregulated DEPs/DEGs.


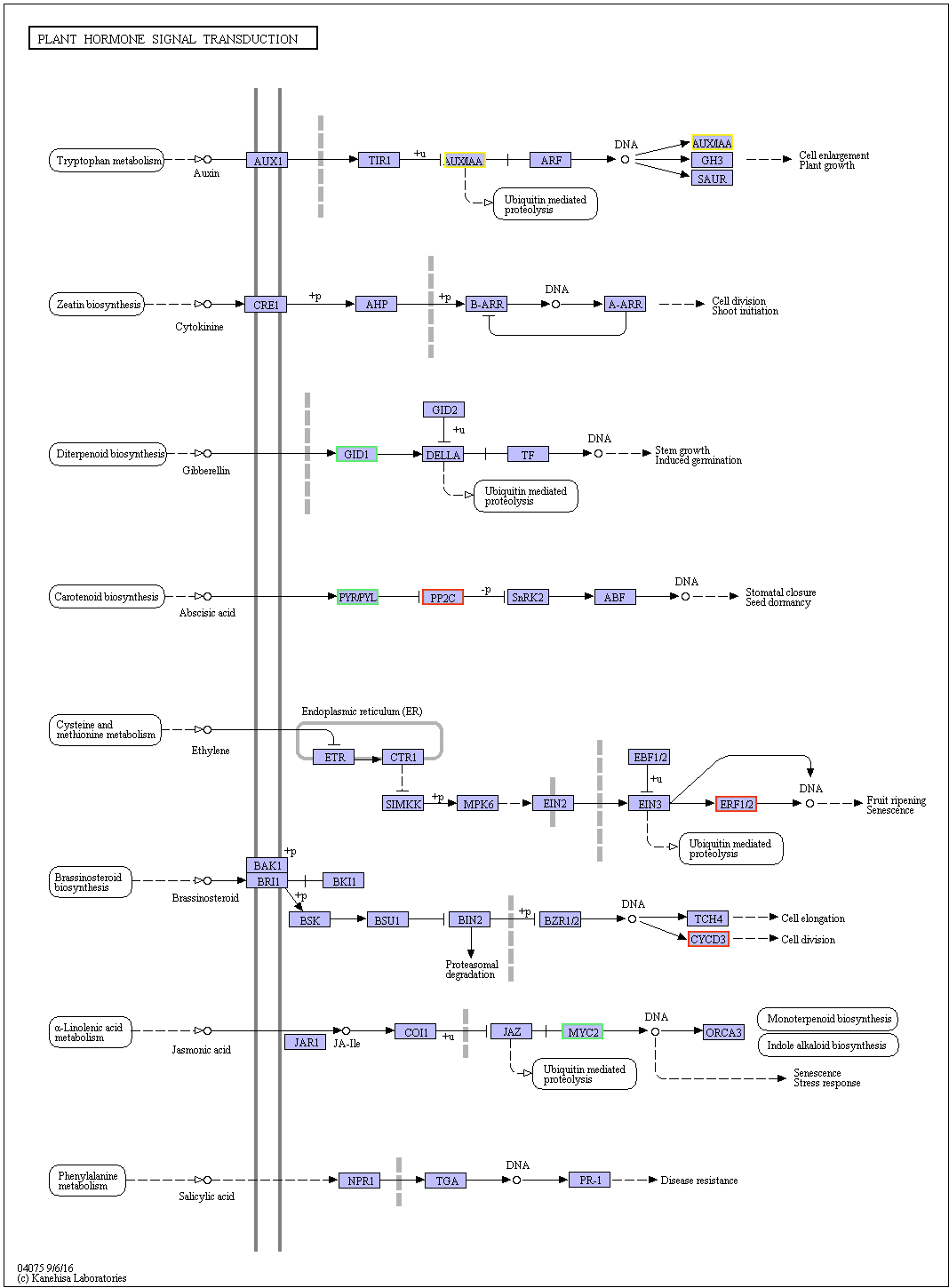


**Fig. S6**  Changes in plant signal transduction pathways after AMF inoculation. The purple annotations represent enriched DEGs. The red boxes indicate upregulated DEGs, the green boxes represent downregulated DEGs, and the yellow boxes indicate DEGs that are both upregulated and downregulated.

**1.2 Supplementary Tables**

**Table S1** GO pathway enrichment of DEPs between AMF and Control groups

| GO_ID | GO_Term | GO_Class | Pvalue |
| --- | --- | --- | --- |
| GO:0071705 | nitrogen compound transport | BP | 0.000850253 |
| GO:0008380 | RNA splicing | BP | 0.002501947 |
| GO:0015698 | inorganic anion transport | BP | 0.002501947 |
| GO:0005215 | transporter activity | MF | 0.003634868 |
| GO:0006811 | ion transport | BP | 0.005379477 |
| GO:0015075 | ion transmembrane transporter activity | MF | 0.008263353 |
| GO:0030529 | intracellular ribonucleoprotein complex | CC | 0.010568698 |
| GO:0003993 | acid phosphatase activity | MF | 0.021214831 |
| GO:0006366 | transcription from RNA polymerase II promoter | BP | 0.021214831 |
| GO:0019825 | oxygen binding | MF | 0.029310882 |
| GO:0008519 | ammonium transmembrane transporter activity | MF | 0.029310882 |
| GO:0015696 | ammonium transport | BP | 0.029310882 |
| GO:0010167 | response to nitrate | BP | 0.029310882 |
| GO:0015706 | nitrate transport | BP | 0.029310882 |
| GO:0015098 | molybdate ion transmembrane transporter activity | MF | 0.029310882 |
| GO:0015689 | molybdate ion transport | BP | 0.029310882 |
| GO:0006659 | phosphatidylserine biosynthetic process | BP | 0.029310882 |
| GO:0004611 | phosphoenolpyruvate carboxykinase activity | MF | 0.032821442 |
| GO:0044428 | nuclear part | CC | 0.035177526 |
| GO:0008324 | cation transmembrane transporter activity | MF | 0.038942953 |
| GO:0015991 | ATP hydrolysis coupled proton transport | BP | 0.03936123 |
| GO:0022613 | ribonucleoprotein complex biogenesis | BP | 0.046347917 |
| GO:0020037 | heme binding | MF | 0.053356386 |
| GO:0006812 | cation transport | BP | 0.057161688 |
| GO:0004612 | phosphoenolpyruvate carboxykinase (ATP) activity | MF | 0.057771512 |
| GO:0005685 | U1 snRNP | CC | 0.057771512 |
| GO:0006376 | mRNA splice site selection | BP | 0.057771512 |
| GO:0006364 | rRNA processing | BP | 0.057771512 |
| GO:0016593 | Cdc73/Paf1 complex | CC | 0.057771512 |
| GO:0006979 | response to oxidative stress | BP | 0.060670148 |
| GO:0010467 | gene expression | BP | 0.073181443 |
| GO:0004601 | peroxidase activity | MF | 0.075905526 |
| GO:0004386 | helicase activity | MF | 0.078160182 |
| GO:0005840 | ribosome | CC | 0.085044255 |
| GO:0005681 | spliceosomal complex | CC | 0.085406294 |
| GO:0008375 | acetylglucosaminyltransferase activity | MF | 0.085406294 |
| GO:0006368 | transcription elongation from RNA polymerase II promoter | BP | 0.085406294 |
| GO:0006396 | RNA processing | BP | 0.086910738 |
| GO:0003735 | structural constituent of ribosome | MF | 0.089291464 |
| GO:0005634 | nucleus | CC | 0.090674902 |
| GO:0097159 | organic cyclic compound binding | MF | 0.098331491 |
| GO:1901363 | heterocyclic compound binding | MF | 0.098331491 |
| GO:0034654 | nucleobase-containing compound biosynthetic process | BP | 0.10459703 |
| GO:0004478 | methionine adenosyltransferase activity | MF | 0.112238943 |
| GO:0006556 | S-adenosylmethionine biosynthetic process | BP | 0.112238943 |
| GO:0006801 | superoxide metabolic process | BP | 0.112238943 |
| GO:0003729 | mRNA binding | MF | 0.112238943 |
| GO:0015078 | hydrogen ion transmembrane transporter activity | MF | 0.114866996 |
| GO:0006094 | gluconeogenesis | BP | 0.138292499 |
| GO:0004550 | nucleoside diphosphate kinase activity | MF | 0.138292499 |
| GO:0006183 | GTP biosynthetic process | BP | 0.138292499 |
| GO:0006228 | UTP biosynthetic process | BP | 0.138292499 |
| GO:0006241 | CTP biosynthetic process | BP | 0.138292499 |
| GO:0016570 | histone modification | BP | 0.138292499 |
| GO:0000272 | polysaccharide catabolic process | BP | 0.163589347 |
| GO:0016161 | beta-amylase activity | MF | 0.163589347 |
| GO:0003860 | 3-hydroxyisobutyryl-CoA hydrolase activity | MF | 0.163589347 |
| GO:0003676 | nucleic acid binding | MF | 0.178048842 |
| GO:0008194 | UDP-glycosyltransferase activity | MF | 0.186594948 |
| GO:0004471 | malate dehydrogenase (decarboxylating) (NAD+) activity | MF | 0.188151234 |
| GO:0034645 | cellular macromolecule biosynthetic process | BP | 0.195658386 |
| GO:0005976 | polysaccharide metabolic process | BP | 0.197328211 |
| GO:0016616 | oxidoreductase activity, acting on the CH-OH group of donors, NAD or NADP as acceptor | MF | 0.197328211 |
| GO:0008964 | phosphoenolpyruvate carboxylase activity | MF | 0.211999291 |
| GO:0030001 | metal ion transport | BP | 0.211999291 |
| GO:0004616 | phosphogluconate dehydrogenase (decarboxylating) activity | MF | 0.211999291 |
| GO:0016760 | cellulose synthase (UDP-forming) activity | MF | 0.211999291 |
| GO:0046872 | metal ion binding | MF | 0.212795802 |
| GO:0016070 | RNA metabolic process | BP | 0.213118445 |
| GO:0009055 | electron carrier activity | MF | 0.219007494 |
| GO:0051287 | NAD binding | MF | 0.219007494 |
| GO:0006099 | tricarboxylic acid cycle | BP | 0.235154048 |
| GO:0015977 | carbon fixation | BP | 0.235154048 |
| GO:0016051 | carbohydrate biosynthetic process | BP | 0.251866927 |
| GO:0016788 | hydrolase activity, acting on ester bonds | MF | 0.256647058 |
| GO:0007034 | vacuolar transport | BP | 0.257635449 |
| GO:0006810 | transport | BP | 0.263780493 |
| GO:0008033 | tRNA processing | BP | 0.279462871 |
| GO:0043565 | sequence-specific DNA binding | MF | 0.279462871 |
| GO:0006950 | response to stress | BP | 0.28924149 |
| GO:0008270 | zinc ion binding | MF | 0.299254134 |
| GO:0006165 | nucleoside diphosphate phosphorylation | BP | 0.306808615 |
| GO:0043933 | macromolecular complex subunit organization | BP | 0.317738971 |
| GO:0090407 | organophosphate biosynthetic process | BP | 0.317738971 |
| GO:0003899 | DNA-directed RNA polymerase activity | MF | 0.321230544 |
| GO:0003779 | actin binding | MF | 0.321230544 |
| GO:0006486 | protein glycosylation | BP | 0.321230544 |
| GO:0017111 | nucleoside-triphosphatase activity | MF | 0.322746455 |
| GO:0016052 | carbohydrate catabolic process | BP | 0.339478174 |
| GO:0009607 | response to biotic stimulus | BP | 0.341206852 |
| GO:0030244 | cellulose biosynthetic process | BP | 0.341206852 |
| GO:0016758 | transferase activity, transferring hexosyl groups | MF | 0.350273251 |
| GO:0019867 | outer membrane | CC | 0.360601326 |
| GO:0044271 | cellular nitrogen compound biosynthetic process | BP | 0.360780089 |
| GO:1901137 | carbohydrate derivative biosynthetic process | BP | 0.361010178 |
| GO:0006091 | generation of precursor metabolites and energy | BP | 0.371683115 |
| GO:0051536 | iron-sulfur cluster binding | MF | 0.379430736 |
| GO:0006952 | defense response | BP | 0.379430736 |
| GO:0008168 | methyltransferase activity | MF | 0.382286603 |
| GO:0004743 | pyruvate kinase activity | MF | 0.397711372 |
| GO:0030955 | potassium ion binding | MF | 0.397711372 |
| GO:0043229 | intracellular organelle | CC | 0.40545023 |
| GO:0055114 | oxidation-reduction process | BP | 0.40733691 |
| GO:1901576 | organic substance biosynthetic process | BP | 0.40783144 |
| GO:0003723 | RNA binding | MF | 0.417555279 |
| GO:0044249 | cellular biosynthetic process | BP | 0.419823801 |
| GO:0034641 | cellular nitrogen compound metabolic process | BP | 0.438080859 |
| GO:0009058 | biosynthetic process | BP | 0.472318911 |
| GO:0003924 | GTPase activity | MF | 0.526008131 |
| GO:0032991 | macromolecular complex | CC | 0.557917424 |
| GO:0050660 | flavin adenine dinucleotide binding | MF | 0.579608031 |
| GO:0006732 | coenzyme metabolic process | BP | 0.650871483 |
| GO:0006412 | translation | BP | 0.659223711 |
| GO:0006807 | nitrogen compound metabolic process | BP | 0.686138799 |
| GO:0009205 | purine ribonucleoside triphosphate metabolic process | BP | 0.699364266 |
| GO:0009150 | purine ribonucleotide metabolic process | BP | 0.715900012 |
| GO:0046128 | purine ribonucleoside metabolic process | BP | 0.715900012 |
| GO:0050662 | coenzyme binding | MF | 0.743074585 |
| GO:0044723 | single-organism carbohydrate metabolic process | BP | 0.744920905 |
| GO:0019637 | organophosphate metabolic process | BP | 0.750771826 |
| GO:0003677 | DNA binding | MF | 0.752816893 |
| GO:1901135 | carbohydrate derivative metabolic process | BP | 0.765900423 |
| GO:0071840 | cellular component organization or biogenesis | BP | 0.768193889 |
| GO:0005488 | binding | MF | 0.783949979 |
| GO:0050896 | response to stimulus | BP | 0.803516183 |
| GO:0046914 | transition metal ion binding | MF | 0.816087973 |
| GO:1901566 | organonitrogen compound biosynthetic process | BP | 0.860626118 |
| GO:0044260 | cellular macromolecule metabolic process | BP | 0.867854508 |
| GO:0016491 | oxidoreductase activity | MF | 0.878305736 |
| GO:0006139 | nucleobase-containing compound metabolic process | BP | 0.904054398 |
| GO:0005622 | intracellular | CC | 0.911340421 |
| GO:0044424 | intracellular part | CC | 0.91654021 |
| GO:0005975 | carbohydrate metabolic process | BP | 0.923977456 |
| GO:0016020 | membrane | CC | 1 |
| GO:0044710 | single-organism metabolic process | BP | 1 |
| GO:0000166 | nucleotide binding | MF | 1 |
| GO:0006096 | glycolytic process | BP | 1 |

**Table S2** GO annotation of up-regulated or down-regulated DEPs

| GO_ID | GO_Term | GO_Class | Pvalue | Up/Down | Number |
| --- | --- | --- | --- | --- | --- |
| GO:0005215 | transporter activity | MF | 3.84E-05 | Up | 6 |
| GO:0071705 | nitrogen compound transport | BP | 9.05E-05 | Up | 2 |
| GO:0006811 | ion transport | BP | 0.000162807 | Up | 5 |
| GO:0006812 | cation transport | BP | 0.001206902 | Up | 4 |
| GO:0020037 | heme binding | MF | 0.001721291 | Up | 5 |
| GO:0008324 | cation transmembrane transporter activity | MF | 0.001829879 | Up | 3 |
| GO:0006810 | transport | BP | 0.002483379 | Up | 8 |
| GO:0006979 | response to oxidative stress | BP | 0.003061769 | Up | 3 |
| GO:0004601 | peroxidase activity | MF | 0.003996851 | Up | 3 |
| GO:0015991 | ATP hydrolysis coupled proton transport | BP | 0.00471177 | Up | 2 |
| GO:0019825 | oxygen binding | MF | 0.009666355 | Up | 1 |
| GO:0008519 | ammonium transmembrane transporter activity | MF | 0.009666355 | Up | 1 |
| GO:0015696 | ammonium transport | BP | 0.009666355 | Up | 1 |
| GO:0010167 | response to nitrate | BP | 0.009666355 | Up | 1 |
| GO:0015706 | nitrate transport | BP | 0.009666355 | Up | 1 |
| GO:0006659 | phosphatidylserine biosynthetic process | BP | 0.009666355 | Up | 1 |
| GO:0015078 | hydrogen ion transmembrane transporter activity | MF | 0.015420227 | Up | 2 |
| GO:0004478 | methionine adenosyltransferase activity | MF | 0.038125971 | Up | 1 |
| GO:0006556 | S-adenosylmethionine biosynthetic process | BP | 0.038125971 | Up | 1 |
| GO:0016491 | oxidoreductase activity | MF | 0.04521055 | Up | 6 |
| GO:0000272 | polysaccharide catabolic process | BP | 0.056657604 | Up | 1 |
| GO:0016161 | beta-amylase activity | MF | 0.056657604 | Up | 1 |
| GO:0050896 | response to stimulus | BP | 0.057527693 | Up | 4 |
| GO:0016052 | carbohydrate catabolic process | BP | 0.058724516 | Up | 2 |
| GO:0030001 | metal ion transport | BP | 0.074843505 | Up | 1 |
| GO:0004616 | phosphogluconate dehydrogenase (decarboxylating) activity | MF | 0.074843505 | Up | 1 |
| GO:0003993 | acid phosphatase activity | MF | 0.074843505 | Up | 1 |
| GO:0006732 | coenzyme metabolic process | BP | 0.077509334 | Up | 2 |
| GO:0016020 | membrane | CC | 0.095943344 | Up | 5 |
| GO:0055114 | oxidation-reduction process | BP | 0.101618316 | Up | 7 |
| GO:0019867 | outer membrane | CC | 0.135860621 | Up | 1 |
| GO:0051536 | iron-sulfur cluster binding | MF | 0.144252952 | Up | 1 |
| GO:0004743 | pyruvate kinase activity | MF | 0.152566383 | Up | 1 |
| GO:0030955 | potassium ion binding | MF | 0.152566383 | Up | 1 |
| GO:0019637 | organophosphate metabolic process | BP | 0.222730113 | Up | 2 |
| GO:0051287 | NAD binding | MF | 0.253779007 | Up | 1 |
| GO:0009055 | electron carrier activity | MF | 0.253779007 | Up | 1 |
| GO:0006096 | glycolytic process | BP | 0.275417351 | Up | 1 |
| GO:0016887 | ATPase activity | MF | 0.303325907 | Up | 1 |
| GO:0000287 | magnesium ion binding | MF | 0.310138808 | Up | 1 |
| GO:0044699 | single-organism process | BP | 0.400893646 | Up | 11 |
| GO:0044710 | single-organism metabolic process | BP | 0.465489243 | Up | 9 |
| GO:0046872 | metal ion binding | MF | 0.532566085 | Up | 4 |
| GO:0005634 | nucleus | CC | 0.553707208 | Up | 1 |
| GO:0097159 | organic cyclic compound binding | MF | 0.733682245 | Up | 11 |
| GO:1901363 | heterocyclic compound binding | MF | 0.733682245 | Up | 11 |
| GO:0016787 | hydrolase activity | MF | 0.782234384 | Up | 4 |
| GO:0005525 | GTP binding | MF | 1 | Up | 1 |
| GO:0008380 | RNA splicing | BP | 0.00112524 | Down | 2 |
| GO:0030529 | intracellular ribonucleoprotein complex | CC | 0.002854522 | Down | 8 |
| GO:0010467 | gene expression | BP | 0.005964227 | Down | 11 |
| GO:0003676 | nucleic acid binding | MF | 0.006829729 | Down | 15 |
| GO:0006366 | transcription from RNA polymerase II promoter | BP | 0.009855272 | Down | 2 |
| GO:0044428 | nuclear part | CC | 0.01221228 | Down | 3 |
| GO:0004611 | phosphoenolpyruvate carboxykinase activity | MF | 0.015443023 | Down | 2 |
| GO:0015098 | molybdate ion transmembrane transporter activity | MF | 0.019644528 | Down | 1 |
| GO:0015689 | molybdate ion transport | BP | 0.019644528 | Down | 1 |
| GO:0022613 | ribonucleoprotein complex biogenesis | BP | 0.022085361 | Down | 2 |
| GO:0034645 | cellular macromolecule biosynthetic process | BP | 0.026221895 | Down | 10 |
| GO:0005840 | ribosome | CC | 0.027849586 | Down | 6 |
| GO:0003735 | structural constituent of ribosome | MF | 0.030999303 | Down | 6 |
| GO:0006396 | RNA processing | BP | 0.032552393 | Down | 3 |
| GO:0004386 | helicase activity | MF | 0.03818731 | Down | 2 |
| GO:0004612 | phosphoenolpyruvate carboxykinase (ATP) activity | MF | 0.038909155 | Down | 1 |
| GO:0005685 | U1 snRNP | CC | 0.038909155 | Down | 1 |
| GO:0006376 | mRNA splice site selection | BP | 0.038909155 | Down | 1 |
| GO:0006364 | rRNA processing | BP | 0.038909155 | Down | 1 |
| GO:0016593 | Cdc73/Paf1 complex | CC | 0.038909155 | Down | 1 |
| GO:0034654 | nucleobase-containing compound biosynthetic process | BP | 0.039979742 | Down | 3 |
| GO:0016070 | RNA metabolic process | BP | 0.043370547 | Down | 5 |
| GO:0044271 | cellular nitrogen compound biosynthetic process | BP | 0.053935855 | Down | 9 |
| GO:0005681 | spliceosomal complex | CC | 0.057801112 | Down | 1 |
| GO:0008375 | acetylglucosaminyltransferase activity | MF | 0.057801112 | Down | 1 |
| GO:0006368 | transcription elongation from RNA polymerase II promoter | BP | 0.057801112 | Down | 1 |
| GO:0008270 | zinc ion binding | MF | 0.073203569 | Down | 4 |
| GO:0005634 | nucleus | CC | 0.075864968 | Down | 4 |
| GO:0006801 | superoxide metabolic process | BP | 0.076327495 | Down | 1 |
| GO:0003729 | mRNA binding | MF | 0.076327495 | Down | 1 |
| GO:0006094 | gluconeogenesis | BP | 0.094495265 | Down | 1 |
| GO:0004550 | nucleoside diphosphate kinase activity | MF | 0.094495265 | Down | 1 |
| GO:0006183 | GTP biosynthetic process | BP | 0.094495265 | Down | 1 |
| GO:0006228 | UTP biosynthetic process | BP | 0.094495265 | Down | 1 |
| GO:0006241 | CTP biosynthetic process | BP | 0.094495265 | Down | 1 |
| GO:0016570 | histone modification | BP | 0.094495265 | Down | 1 |
| GO:0008194 | UDP-glycosyltransferase activity | MF | 0.097432679 | Down | 2 |
| GO:1901576 | organic substance biosynthetic process | BP | 0.099682365 | Down | 12 |
| GO:0097159 | organic cyclic compound binding | MF | 0.102723869 | Down | 26 |
| GO:1901363 | heterocyclic compound binding | MF | 0.102723869 | Down | 26 |
| GO:0034641 | cellular nitrogen compound metabolic process | BP | 0.109992117 | Down | 12 |
| GO:0003860 | 3-hydroxyisobutyryl-CoA hydrolase activity | MF | 0.112311254 | Down | 1 |
| GO:0044260 | cellular macromolecule metabolic process | BP | 0.117038107 | Down | 15 |
| GO:0004471 | malate dehydrogenase (decarboxylating) (NAD+) activity | MF | 0.129782167 | Down | 1 |
| GO:0016051 | carbohydrate biosynthetic process | BP | 0.136172808 | Down | 2 |
| GO:0032991 | macromolecular complex | CC | 0.146191577 | Down | 9 |
| GO:0008964 | phosphoenolpyruvate carboxylase activity | MF | 0.14691458 | Down | 1 |
| GO:0003993 | acid phosphatase activity | MF | 0.14691458 | Down | 1 |
| GO:0016760 | cellulose synthase (UDP-forming) activity | MF | 0.14691458 | Down | 1 |
| GO:0043229 | intracellular organelle | CC | 0.147790926 | Down | 10 |
| GO:0006099 | tricarboxylic acid cycle | BP | 0.16371495 | Down | 1 |
| GO:0015977 | carbon fixation | BP | 0.16371495 | Down | 1 |
| GO:0043933 | macromolecular complex subunit organization | BP | 0.177681692 | Down | 2 |
| GO:0007034 | vacuolar transport | BP | 0.180189608 | Down | 1 |
| GO:0008033 | tRNA processing | BP | 0.196344771 | Down | 1 |
| GO:0043565 | sequence-specific DNA binding | MF | 0.196344771 | Down | 1 |
| GO:0016758 | transferase activity, transferring hexosyl groups | MF | 0.199126673 | Down | 2 |
| GO:1901137 | carbohydrate derivative biosynthetic process | BP | 0.206347374 | Down | 2 |
| GO:0044249 | cellular biosynthetic process | BP | 0.21812587 | Down | 11 |
| GO:0008168 | methyltransferase activity | MF | 0.220873101 | Down | 2 |
| GO:0003899 | DNA-directed RNA polymerase activity | MF | 0.227720885 | Down | 1 |
| GO:0003779 | actin binding | MF | 0.227720885 | Down | 1 |
| GO:0006486 | protein glycosylation | BP | 0.227720885 | Down | 1 |
| GO:0009607 | response to biotic stimulus | BP | 0.242953692 | Down | 1 |
| GO:0030244 | cellulose biosynthetic process | BP | 0.242953692 | Down | 1 |
| GO:0017111 | nucleoside-triphosphatase activity | MF | 0.243232445 | Down | 3 |
| GO:0006412 | translation | BP | 0.268371488 | Down | 6 |
| GO:0006952 | defense response | BP | 0.27253761 | Down | 1 |
| GO:0046872 | metal ion binding | MF | 0.285585496 | Down | 9 |
| GO:0006139 | nucleobase-containing compound metabolic process | BP | 0.306338798 | Down | 6 |
| GO:0003723 | RNA binding | MF | 0.311677213 | Down | 4 |
| GO:0005622 | intracellular | CC | 0.321806186 | Down | 12 |
| GO:0016788 | hydrolase activity, acting on ester bonds | MF | 0.330830534 | Down | 2 |
| GO:0044424 | intracellular part | CC | 0.373493803 | Down | 11 |
| GO:0046914 | transition metal ion binding | MF | 0.379694373 | Down | 5 |
| GO:0003924 | GTPase activity | MF | 0.392185371 | Down | 1 |
| GO:0003677 | DNA binding | MF | 0.429092758 | Down | 3 |
| GO:0050660 | flavin adenine dinucleotide binding | MF | 0.438932138 | Down | 1 |
| GO:0071840 | cellular component organization or biogenesis | BP | 0.446576069 | Down | 3 |
| GO:0009055 | electron carrier activity | MF | 0.450054628 | Down | 1 |
| GO:0051287 | NAD binding | MF | 0.450054628 | Down | 1 |
| GO:0005488 | binding | MF | 0.46479404 | Down | 35 |
| GO:0044237 | cellular metabolic process | BP | 0.475667892 | Down | 18 |
| GO:0006165 | nucleoside diphosphate phosphorylation | BP | 0.531560899 | Down | 1 |
| GO:0044267 | cellular protein metabolic process | BP | 0.543333455 | Down | 10 |
| GO:0005509 | calcium ion binding | MF | 0.601150658 | Down | 1 |
| GO:0044238 | primary metabolic process | BP | 0.604621144 | Down | 21 |

**Table S3** The subcellular localization of DEPs

| Protein_id | Gene | E_value | Subcellular_localization |
| --- | --- | --- | --- |
| Prot_16073 | Cluster-12938.43798_orf1 | 2.00E-91 | nucleus protein |
| Prot_23947 | Cluster-12938.18892_orf1 | 0 | cytoplasm protein |
| Prot_25370 | Cluster-12938.34382_orf2 | 0 | cell membrane protein |
| Prot_41677 | Gene.11934::Cluster-12938.31249::g.11934::m.11934 | 0 | cytoplasm protein |
| Prot_2643 | Cluster-12938.39999_orf1 | 4.00E-94 | cell wall protein |
| Prot_1019 | Cluster-12938.38314_orf2 | 1.00E-10 | mitochondrion protein |
| Prot_313 | Cluster-12938.35945_orf1 | 1.00E-139 | cell membrane protein |
| Prot_40909 | Gene.10976::Cluster-12938.26777::g.10976::m.10976 | 1.00E-68 | cytoplasm protein |
| Prot_25920 | Cluster-12938.38174_orf1 | 5.00E-46 | chloroplast protein |
| Prot_32061 | Cluster-12938.25251_orf1 | 1.00E-134 | mitochondrion protein |
| Prot_4374 | Cluster-12938.33531_orf1 | 5.00E-32 | cytoplasm protein |
| Prot_13437 | Cluster-12938.34293_orf1 | 2.00E-63 | cell membrane protein |
| Prot_2313 | Cluster-12938.18635_orf1 | 1.00E-99 | nucleus protein |
| Prot_8754 | Cluster-12938.34965_orf1 | 1.00E-117 | cell membrane protein |
| Prot_9192 | Cluster-12938.30424_orf2 | 0 | chloroplast protein |
| Prot_42426 | Gene.15311::Cluster-12938.35581::g.15311::m.15311 | 6.00E-82 | cell wall protein |
| Prot_36790 | Cluster-12938.62157_orf1 | 3.00E-15 | peroxisome protein |
| Prot_3881 | Cluster-12938.4535_orf1 | 2.00E-12 | peroxisome protein |
| Prot_22582 | Cluster-12938.41528_orf1 | 1.00E-10 | peroxisome protein |
| Prot_8355 | Cluster-12938.22308_orf1 | 5.00E-09 | cell membrane protein |
| Prot_16138 | Cluster-12938.34013_orf1 | 1.00E-27 | nucleus protein |
| Prot_12151 | Cluster-12938.27406_orf1 | 1.00E-126 | chloroplast protein |
| Prot_35595 | Cluster-12938.37583_orf1 | 3.00E-37 | cell membrane protein |
| Prot_11490 | Cluster-12938.63126_orf1 | 6.00E-33 | chloroplast protein |
| Prot_9741 | Cluster-12938.10710_orf1 | 5.00E-21 | vacuole protein |
| Prot_627 | Cluster-12938.36694_orf2 | 1.00E-25 | cytoplasm protein |
| Prot_36069 | Cluster-12938.35240_orf1 | 8.00E-11 | nucleus protein |
| Prot_6267 | Cluster-12938.22630_orf1 | 8.00E-09 | mitochondrion protein |
| Prot_46011 | Gene.6450::Cluster-12938.56115::g.6450::m.6450 | 4.00E-66 | cell wall protein |
| Prot_5479 | Cluster-12938.33150_orf1 | 1.00E-56 | cytoplasm protein |
| Prot_29558 | Cluster-12938.56767_orf1 | 9.00E-32 | cell wall protein |
| Prot_34469 | Cluster-12938.28366_orf1 | 0 | cell membrane protein |
| Prot_9191 | Cluster-12938.30424_orf1 | 1.00E-39 | chloroplast protein |
| Prot_12373 | Cluster-12938.34101_orf1 | 6.00E-25 | cell membrane protein |
| Prot_20832 | Cluster-12938.27825_orf2 | 1.00E-58 | cytoplasm protein |
| Prot_21162 | Cluster-12938.37609_orf2 | 9.00E-09 | vacuole protein |
| Prot_14626 | Cluster-12938.41151_orf1 | 1.00E-125 | cell wall protein |
| Prot_7387 | Cluster-1790.0_orf1 | 4.00E-31 | chloroplast protein |
| Prot_42109 | Gene.11351::Cluster-12938.33815::g.11351::m.11351 | 1.00E-112 | vacuole protein |
| Prot_5767 | Cluster-12938.26811_orf1 | 2.00E-33 | cell membrane protein |
| Prot_9748 | Cluster-12938.27314_orf1 | 5.00E-11 | nucleus protein |
| Prot_18745 | Cluster-12938.43249_orf1 | 1.00E-165 | cytoplasm protein |
| Prot_1629 | Cluster-12938.31273_orf2 | 2.00E-97 | chloroplast protein |
| Prot_12106 | Cluster-12938.48030_orf1 | 6.00E-16 | cytoplasm protein |
| Prot_23851 | Cluster-12938.51771_orf1 | 0 | cytoplasm protein |
| Prot_18828 | Cluster-12938.27742_orf1 | 1.00E-14 | cell membrane protein |
| Prot_14975 | Cluster-12938.30795_orf1 | 8.00E-72 | nucleus protein |
| Prot_16905 | Cluster-12938.29585_orf1 | 2.00E-12 | cell membrane protein |
| Prot_11057 | Cluster-12938.34183_orf2 | 9.00E-29 | nucleus protein |
| Prot_42220 | Gene.2531::Cluster-12938.34450::g.2531::m.2531 | 9.00E-76 | nucleus protein |
| Prot_41623 | Gene.8436::Cluster-12938.30954::g.8436::m.8436 | 2.00E-08 | plastid protein |
| Prot_47939 | Gene.1357::Cluster-12938.8847::g.1357::m.1357 | 1.00E-36 | plastid protein |
| Prot_1427 | Cluster-12938.21086_orf1 | 1.00E-167 | chloroplast protein |
| Prot_33236 | Cluster-12938.32660_orf1 | 6.00E-42 | nucleus protein |
| Prot_12345 | Cluster-12938.62269_orf3 | 2.00E-09 | vacuole protein |
| Prot_28580 | Cluster-12938.48487_orf1 | 1.00E-09 | extracell protein |
| Prot_226 | Cluster-12938.54138_orf1 | 1.00E-132 | cell wall protein |
| Prot_9275 | Cluster-12938.16621_orf1 | 4.00E-25 | nucleus protein |
| Prot_15365 | Cluster-12938.41055_orf1 | 5.00E-40 | cytoplasm protein |
| Prot_35360 | Cluster-12938.26935_orf1 | 3.00E-84 | chloroplast protein |
| Prot_17013 | Cluster-12938.38427_orf1 | 4.00E-52 | cytoplasm protein |
| Prot_11505 | Cluster-12938.47542_orf1 | 2.00E-15 | chloroplast protein |
| Prot_10840 | Cluster-12938.23496_orf2 | 3.00E-80 | cytoplasm protein |
| Prot_34105 | Cluster-12938.37888_orf1 | 4.00E-06 | vacuole protein |
| Prot_43056 | Gene.8547::Cluster-12938.39262::g.8547::m.8547 | 6.00E-80 | chloroplast protein |
| Prot_33355 | Cluster-12938.41356_orf1 | 2.00E-08 | nucleus protein |
| Prot_12605 | Cluster-12938.27192_orf1 | 2.00E-13 | nucleus protein |
| Prot_32799 | Cluster-12938.36366_orf2 | 2.00E-16 | cytoplasm protein |
| Prot_15221 | Cluster-12938.41449_orf2 | 1.00E-05 | nucleus protein |
| Prot_20167 | Cluster-12938.34244_orf1 | 2.00E-05 | nucleus protein |
| Prot_45361 | Gene.2332::Cluster-12938.52110::g.2332::m.2332 | 1.00E-154 | cytoplasm protein |
| Prot_10550 | Cluster-12938.29417_orf1 | 7.00E-09 | chloroplast protein |
| Prot_40240 | Gene.12113::Cluster-12938.22679::g.12113::m.12113 | 4.00E-07 | mitochondrion protein |
| Prot_15425 | Cluster-12938.16526_orf1 | 1.00E-21 | nucleus protein |
| Prot_11345 | Cluster-12938.49717_orf1 | 2.00E-22 | mitochondrion protein |
| Prot_568 | Cluster-12938.39633_orf2 | 1.00E-07 | nucleus protein |
| Prot_16908 | Cluster-12938.29589_orf1 | 2.00E-43 | vacuole protein |
| Prot_26677 | Cluster-12938.48294_orf1 | 3.00E-11 | cytoplasm protein |

**Table S4** KEGG enrichment of DEP_S_

| MapID | MapTitle | Pvalue | Diff Proteins with pathway |
| --- | --- | --- | --- |
| map03010 | Ribosome | 0.004479691 | 27 |
| map03008 | Ribosome biogenesis in eukaryotes | 0.005810068 | 8 |
| map00910 | Nitrogen metabolism | 0.012534813 | 4 |
| map03410 | Base excision repair | 0.012534813 | 4 |
| map03430 | Mismatch repair | 0.013978798 | 5 |
| map03420 | Nucleotide excision repair | 0.028901887 | 6 |
| map04136 | Autophagy - other | 0.068468127 | 3 |
| map03030 | DNA replication | 0.099522395 | 5 |
| map00514 | Other types of O-glycan biosynthesis | 0.245791849 | 1 |
| map00100 | Steroid biosynthesis | 0.258766885 | 2 |
| map00195 | Photosynthesis | 0.258766885 | 2 |
| map03040 | Spliceosome | 0.261165141 | 15 |
| map04075 | Plant hormone signal transduction | 0.274915764 | 4 |
| map00300 | Lysine biosynthesis | 0.293429028 | 2 |
| map03020 | RNA polymerase | 0.293429028 | 2 |
| map00240 | Pyrimidine metabolism | 0.334295161 | 7 |
| map00920 | Sulfur metabolism | 0.470107294 | 3 |
| map00902 | Monoterpenoid biosynthesis | 0.482524745 | 1 |
| map04016 | MAPK signaling pathway - plant | 0.52507575 | 4 |
| map02010 | ABC transporters | 0.529084215 | 1 |
| map00940 | Phenylpropanoid biosynthesis | 0.546149875 | 7 |
| map00640 | Propanoate metabolism | 0.568896169 | 4 |
| map00780 | Biotin metabolism | 0.571472691 | 1 |
| map00750 | Vitamin B6 metabolism | 0.6100622 | 1 |
| map00941 | Flavonoid biosynthesis | 0.6100622 | 1 |
| map03018 | RNA degradation | 0.633381814 | 8 |
| map03013 | RNA transport | 0.666321687 | 14 |
| map00460 | Cyanoamino acid metabolism | 0.685668064 | 2 |
| map03060 | Protein export | 0.698905943 | 2 |
| map03015 | mRNA surveillance pathway | 0.735105253 | 9 |
| map00561 | Glycerolipid metabolism | 0.740701848 | 3 |
| map00230 | Purine metabolism | 0.745955283 | 10 |
| map00270 | Cysteine and methionine metabolism | 0.863211769 | 7 |
| map00620 | Pyruvate metabolism | 0.977433711 | 10 |
| map00020 | Citrate cycle (TCA cycle) | 1 | 7 |
| map00970 | Aminoacyl-tRNA biosynthesis | 1 | 6 |
| map00261 | Monobactam biosynthesis | 1 | 1 |
| map00945 | Stilbenoid, diarylheptanoid and gingerol biosynthesis | 1 | 1 |
| map03440 | Homologous recombination | 1 | 1 |
| map00280 | Valine, leucine and isoleucine degradation | 1 | 4 |
| map00510 | N-Glycan biosynthesis | 1 | 2 |
| map04120 | Ubiquitin mediated proteolysis | 1 | 4 |

**Table S5** A domain analysis of the DEPs

| IPRID | IPRTitle | Pvalue |
| --- | --- | --- |
| IPR001223 | Glycoside hydrolase, family 18, catalytic domain | 0.000896094 |
| IPR025886 | Phloem protein 2-like | 0.005166533 |
| IPR000425 | Major intrinsic protein | 0.007572898 |
| IPR008963 | Purple acid phosphatase-like, N-terminal | 0.01703981 |
| IPR015914 | Purple acid phosphatase, N-terminal | 0.01703981 |
| IPR004160 | Translation elongation factor EFTu/EF1A, C-terminal | 0.022276381 |
| IPR009001 | Translation elongation factor EF1A/initiation factor IF2gamma, C-terminal | 0.028083512 |
| IPR000684 | RNA polymerase II, heptapeptide repeat, eukaryotic | 0.030038347 |
| IPR000692 | Fibrillarin | 0.030038347 |
| IPR000894 | Ribulose bisphosphate carboxylase small chain, domain | 0.030038347 |
| IPR000971 | Globin | 0.030038347 |
| IPR001931 | Ribosomal protein S21e | 0.030038347 |
| IPR002379 | ATPase, F0/V0 complex, subunit C | 0.030038347 |
| IPR004098 | Prp18 | 0.030038347 |
| IPR004139 | Glycosyl transferase, family 13 | 0.030038347 |
| IPR004277 | Phosphatidyl serine synthase | 0.030038347 |
| IPR004977 | Ribosomal protein S25 | 0.030038347 |
| IPR005000 | Aldehyde-lyase domain | 0.030038347 |
| IPR007073 | RNA polymerase Rpb1, domain 7 | 0.030038347 |
| IPR007075 | RNA polymerase Rpb1, domain 6 | 0.030038347 |
| IPR007081 | RNA polymerase Rpb1, domain 5 | 0.030038347 |
| IPR007656 | Zein-binding domain | 0.030038347 |
| IPR008509 | Protein of unknown function DUF791 | 0.030038347 |
| IPR012418 | CAP160 | 0.030038347 |
| IPR012562 | GUCT | 0.030038347 |
| IPR013951 | Histone deacetylation protein Rxt3 | 0.030038347 |
| IPR014813 | Guanine nucleotide-binding protein-like 3, N-terminal domain | 0.030038347 |
| IPR014906 | Pre-mRNA processing factor 4 (PRP4)-like | 0.030038347 |
| IPR016605 | Transporter, high affinity nitrate, Nar2 | 0.030038347 |
| IPR018501 | DDT domain superfamily | 0.030038347 |
| IPR018999 | RNA helicase UPF1, UPF2-interacting domain | 0.030038347 |
| IPR019098 | Histone chaperone domain CHZ | 0.030038347 |
| IPR019167 | Topoisomerase II-associated protein PAT1 | 0.030038347 |
| IPR021475 | Protein of unknown function DUF3128 | 0.030038347 |
| IPR024041 | Ammonium transporter AmtB-like | 0.030038347 |
| IPR028942 | WHIM1 domain | 0.030038347 |
| IPR031692 | EH domain-containing protein, N-terminal | 0.030038347 |
| IPR031722 | Coilin, N-terminal domain | 0.030038347 |
| IPR031985 | Domain of unknown function DUF4787 | 0.030038347 |
| IPR032319 | Polyribonucleotide 5'-hydroxyl-kinase Clp1, P-loop domain | 0.030038347 |
| IPR032324 | Clp1, N-terminal beta-sandwich domain | 0.030038347 |
| IPR003245 | Plastocyanin-like | 0.041257233 |
| IPR004000 | Actin-like | 0.048552065 |
| IPR002016 | Haem peroxidase, plant/fungal/bacterial | 0.054869043 |
| IPR001878 | Zinc finger, CCHC-type | 0.056273806 |
| IPR000261 | EPS15 homology (EH) | 0.0591806 |
| IPR000988 | Ribosomal protein L24e-related | 0.0591806 |
| IPR001272 | Phosphoenolpyruvate carboxykinase, ATP-utilising | 0.0591806 |
| IPR001748 | G10 protein | 0.0591806 |
| IPR001976 | Ribosomal protein S24e | 0.0591806 |
| IPR002818 | ThiJ/PfpI | 0.0591806 |
| IPR003657 | DNA-binding WRKY | 0.0591806 |
| IPR004882 | LUC7-related | 0.0591806 |
| IPR005481 | Carbamoyl-phosphate synthase, large subunit, N-terminal | 0.0591806 |
| IPR007083 | RNA polymerase Rpb1, domain 4 | 0.0591806 |
| IPR007133 | RNA polymerase II-associated, Paf1 | 0.0591806 |
| IPR007822 | Lanthionine synthetase C-like | 0.0591806 |
| IPR008195 | Ribosomal protein L34Ae | 0.0591806 |
| IPR008429 | Cleft lip and palate transmembrane 1 | 0.0591806 |
| IPR011008 | Dimeric alpha-beta barrel | 0.0591806 |
| IPR013097 | Stress responsive alpha-beta barrel | 0.0591806 |
| IPR013915 | Pre-mRNA-splicing factor 19 | 0.0591806 |
| IPR014044 | CAP domain | 0.0591806 |
| IPR018545 | Btz domain | 0.0591806 |
| IPR022755 | Zinc finger, double-stranded RNA binding | 0.0591806 |
| IPR023801 | Histone deacetylase domain | 0.0591806 |
| IPR025756 | MYB-CC type transcription factor, LHEQLE-containing domain | 0.0591806 |
| IPR032867 | DYW domain | 0.0591806 |
| IPR004161 | Translation elongation factor EFTu/EF1A, domain 2 | 0.081689625 |
| IPR009000 | Translation elongation/initiation factor/Ribosomal, beta-barrel | 0.081689625 |
| IPR013781 | Glycoside hydrolase, catalytic domain | 0.081689625 |
| IPR000467 | G-patch domain | 0.087453306 |
| IPR001357 | BRCT domain | 0.087453306 |
| IPR001424 | Superoxide dismutase, copper/zinc binding domain | 0.087453306 |
| IPR004201 | CDC48, domain 2 | 0.087453306 |
| IPR004294 | Carotenoid oxygenase | 0.087453306 |
| IPR005117 | Nitrite/sulphite reductase, hemoprotein beta-component, ferrodoxin-like | 0.087453306 |
| IPR005633 | Ribosomal protein L23/L25, N-terminal | 0.087453306 |
| IPR006067 | Nitrite/sulphite reductase 4Fe-4S domain | 0.087453306 |
| IPR006121 | Heavy metal-associated domain, HMA | 0.087453306 |
| IPR011659 | WD40-like Beta Propeller | 0.087453306 |
| IPR012301 | Malic enzyme, N-terminal | 0.087453306 |
| IPR013057 | Amino acid transporter, transmembrane | 0.087453306 |
| IPR013810 | Ribosomal protein S5, N-terminal | 0.087453306 |
| IPR015415 | Vps4 oligomerisation, C-terminal | 0.087453306 |
| IPR016185 | Pre-ATP-grasp fold | 0.087453306 |
| IPR022309 | Ribosomal protein S8e/ribosomal biogenesis NSA2 | 0.087453306 |
| IPR015813 | Pyruvate/Phosphoenolpyruvate kinase | 0.100223415 |
| IPR000184 | Bacterial surface antigen (D15) | 0.11488223 |
| IPR002097 | Profilin/allergen | 0.11488223 |
| IPR002136 | Ribosomal protein L4/L1e | 0.11488223 |
| IPR002715 | Nascent polypeptide-associated complex NAC | 0.11488223 |
| IPR003338 | CDC48, N-terminal subdomain | 0.11488223 |
| IPR004095 | TGS | 0.11488223 |
| IPR005455 | Profilin, plant | 0.11488223 |
| IPR009010 | Aspartate decarboxylase-like fold | 0.11488223 |
| IPR011684 | KIP1-like | 0.11488223 |
| IPR012474 | Frigida-like | 0.11488223 |
| IPR013025 | Ribosomal protein L25/L23 | 0.11488223 |
| IPR018105 | Translationally controlled tumour protein | 0.11488223 |
| IPR022628 | S-adenosylmethionine synthetase, N-terminal | 0.11488223 |
| IPR022629 | S-adenosylmethionine synthetase, central domain | 0.11488223 |
| IPR022630 | S-adenosylmethionine synthetase, C-terminal | 0.11488223 |
| IPR023798 | Ribosomal protein S7 domain | 0.11488223 |
| IPR011032 | GroES-like | 0.119787611 |
| IPR013154 | Alcohol dehydrogenase GroES-like | 0.119787611 |
| IPR002885 | Pentatricopeptide repeat | 0.129897637 |
| IPR003959 | ATPase, AAA-type, core | 0.137666799 |
| IPR000504 | RNA recognition motif domain | 0.138315376 |
| IPR017853 | Glycoside hydrolase, superfamily | 0.140197402 |
| IPR000192 | Aminotransferase, class V/Cysteine desulfurase | 0.14149238 |
| IPR001564 | Nucleoside diphosphate kinase | 0.14149238 |
| IPR002490 | ATPase, V0/A0 complex subunit a | 0.14149238 |
| IPR004152 | GAT | 0.14149238 |
| IPR005479 | Carbamoyl-phosphate synthetase large subunit-like, ATP-binding domain | 0.14149238 |
| IPR006073 | GTP binding domain | 0.14149238 |
| IPR008801 | Rapid ALkalinization Factor | 0.14149238 |
| IPR013078 | Histidine phosphatase superfamily, clade-1 | 0.14149238 |
| IPR029154 | 3-hydroxyisobutyrate dehydrogenase, NAD-binding domain | 0.14149238 |
| IPR000597 | Ribosomal protein L3 | 0.167308028 |
| IPR001048 | Aspartate/glutamate/uridylate kinase | 0.167308028 |
| IPR001554 | Glycoside hydrolase, family 14 | 0.167308028 |
| IPR003029 | Ribosomal protein S1, RNA-binding domain | 0.167308028 |
| IPR012676 | TGS-like | 0.167308028 |
| IPR032259 | Enoyl-CoA hydratase/isomerase, HIBYL-CoA-H type | 0.167308028 |
| IPR001279 | Beta-lactamase-like | 0.192352732 |
| IPR012302 | Malic enzyme, NAD-binding | 0.192352732 |
| IPR001005 | SANT/Myb domain | 0.216649353 |
| IPR001179 | Peptidyl-prolyl cis-trans isomerase, FKBP-type, domain | 0.216649353 |
| IPR001360 | Glycoside hydrolase, family 1 | 0.216649353 |
| IPR005150 | Cellulose synthase | 0.216649353 |
| IPR006115 | 6-phosphogluconate dehydrogenase, NADP-binding | 0.216649353 |
| IPR016140 | Bifunctional inhibitor/plant lipid transfer protein/seed storage helical domain | 0.216649353 |
| IPR021135 | Phosphoenolpyruvate carboxylase | 0.216649353 |
| IPR021131 | Ribosomal protein L18e/L15P | 0.240220083 |
| IPR022812 | Dynamin | 0.240220083 |
| IPR004014 | ATPase, P-type cation-transporter, N-terminal | 0.263086458 |
| IPR005024 | Snf7 | 0.263086458 |
| IPR018316 | Tubulin/FtsZ, 2-layer sandwich domain | 0.263086458 |
| IPR019734 | Tetratricopeptide repeat | 0.263086458 |
| IPR000449 | Ubiquitin-associated/translation elongation factor EF1B, N-terminal | 0.285269381 |
| IPR012675 | Beta-grasp domain | 0.285269381 |
| IPR015940 | Ubiquitin-associated/translation elongation factor EF1B, N-terminal, eukaryote | 0.285269381 |
| IPR003593 | AAA+ ATPase domain | 0.293563601 |
| IPR000916 | Bet v I domain | 0.327665415 |
| IPR004159 | Putative S-adenosyl-L-methionine-dependent methyltransferase | 0.327665415 |
| IPR009057 | Homeodomain-like | 0.327665415 |
| IPR000571 | Zinc finger, CCCH-type | 0.347917322 |
| IPR003008 | Tubulin/FtsZ, GTPase domain | 0.347917322 |
| IPR001680 | WD40 repeat | 0.361307779 |
| IPR008250 | ATPase, P-type, ATPase-associated domain | 0.367563402 |
| IPR012340 | Nucleic acid-binding, OB-fold | 0.386621654 |
| IPR016027 | Nucleic acid-binding, OB-fold-like | 0.386621654 |
| IPR012951 | Berberine/berberine-like | 0.405109544 |
| IPR015793 | Pyruvate kinase, barrel | 0.405109544 |
| IPR023753 | Pyridine nucleotide-disulphide oxidoreductase, FAD/NAD(P)-binding domain | 0.405109544 |
| IPR032799 | Xylanase inhibitor, C-terminal | 0.405109544 |
| IPR032861 | Xylanase inhibitor, N-terminal | 0.405109544 |
| IPR018108 | Mitochondrial substrate/solute carrier | 0.423044022 |
| IPR005123 | Oxoglutarate/iron-dependent dioxygenase | 0.440441539 |
| IPR006094 | FAD linked oxidase, N-terminal | 0.457318062 |
| IPR004088 | K Homology domain, type 1 | 0.473689083 |
| IPR015424 | Pyridoxal phosphate-dependent transferase, major domain | 0.473689083 |
| IPR004843 | Metallophosphoesterase domain | 0.504974323 |
| IPR013149 | Alcohol dehydrogenase, C-terminal | 0.504974323 |
| IPR002048 | Calcium-binding EF-hand | 0.575340894 |
| IPR003439 | ABC transporter-like | 0.588173498 |
| IPR002213 | UDP-glucuronosyl/UDP-glucosyltransferase | 0.600620988 |

**Table S6** Analysis of the correlation between the expression levels of differentially expressed genes and differentially expressed proteins.

| **Gene_id** | **Expression** | **Diff_gene** | **Prot_id** | **Expression** | **Diff_prot** | **correlation** |
| --- | --- | --- | --- | --- | --- | --- |
| Cluster-8378.0 | 6.8361 | _ | Prot_50745 | 0.10230284 | _ | positive |
| Cluster-7518.0 | 1.1783 | _ | Prot_50499 | 0.242512082 | _ | positive |
| Cluster-11574.0 | 1.352 | _ | Prot_15282 | 0.273565687 | _ | positive |
| Cluster-9888.0 | 1.0422 | + | Prot_51181 | 0.074685818 | _ | negative |
| Cluster-7315.0 | 0.63412 | + | Prot_50438 | 0.046328127 | + | positive |
| Cluster-6699.0 | 0.67422 | + | Prot_5694 | 0.025086786 | + | positive |
| Cluster-4700.0 | 0.89724 | _ | Prot_3294 | -0.030190709 | _ | positive |
| Cluster-7077.0 | 1.7911 | _ | Prot_10670 | 0.337937225 | + | negative |
| Cluster-9919.0 | 0.58653 | _ | Prot_23749 | -0.170677385 | _ | positive |
| Cluster-2594.0 | 0.9413 | + | Prot_28958 | -0.002053127 | _ | negative |
| Cluster-577.0 | 0.49713 | _ | Prot_33993 | -0.065163596 | _ | positive |
| Cluster-4423.0 | 2.2441 | _ | Prot_36585 | 0.032602626 | + | negative |
| Cluster-328.0 | 3.184 | + | Prot_49475 | -0.000909453 | _ | negative |
| Cluster-470.0 | 3.8014 | _ | Prot_13425 | 0.140845418 | + | negative |
| Cluster-1369.0 | 0.67043 | _ | Prot_48332 | -0.255257055 | _ | positive |
| Cluster-389.0 | 0.41176 | + | Prot_18927 | -0.152749858 | _ | negative |
| Cluster-360.0 | 0.038822 | + | Prot_49551 | -0.005292281 | _ | negative |
| Cluster-12918.0 | 0.62354 | + | Prot_27558 | -0.073170214 | _ | negative |
| Cluster-546.0 | 3.6171 | + | Prot_49976 | 0.200011804 | _ | negative |
| Cluster-4029.0 | 0.079875 | _ | Prot_19593 | 0.183646078 | _ | positive |
| Cluster-9614.0 | 0.52636 | + | Prot_51107 | 0.137916765 | _ | negative |
| Cluster-366.0 | 0.84727 | _ | Prot_4389 | 0.130794202 | _ | positive |
| Cluster-539.0 | 3.429 | + | Prot_49958 | 0.08450778 | + | positive |
| Cluster-246.0 | 4.2603 | _ | Prot_20209 | 0.131707847 | + | negative |
| Cluster-10376.0 | 0.81803 | _ | Prot_37775 | 0.094855207 | _ | positive |
| Cluster-386.0 | 7.1876 | _ | Prot_49613 | 0.097779719 | _ | positive |
| Cluster-173.0 | 3.6089 | + | Prot_49156 | 0.103002263 | + | positive |
| Cluster-10317.0 | 0.54762 | + | Prot_31970 | 0.428859662 | + | positive |
| Cluster-8243.0 | 1.9059 | _ | Prot_50709 | 0.134324265 | _ | positive |
| Cluster-2525.0 | 1.4312 | + | Prot_49310 | 0.064833711 | _ | negative |
| Cluster-205.0 | 2.2208 | + | Prot_49200 | 0.010044041 | _ | negative |
| Cluster-6959.0 | 1.0616 | _ | Prot_50343 | 0.357834988 | _ | positive |
| Cluster-490.0 | 0.81804 | + | Prot_49840 | 0.182042903 | _ | negative |
| Cluster-5075.0 | 1.7796 | + | Prot_49882 | 0.078763674 | _ | negative |
| Cluster-5931.0 | 1.6126 | _ | Prot_5119 | 0.03356602 | + | negative |
| Cluster-11956.0 | 0.52861 | _ | Prot_15574 | 0.017741257 | + | negative |
| Cluster-6500.0 | 3.8942 | _ | Prot_29293 | 0.201700143 | + | negative |
| Cluster-10306.0 | 2.2274 | _ | Prot_37756 | 0.350849364 | _ | positive |
| Cluster-160.0 | 1.8544 | + | Prot_48922 | 0.044032299 | + | positive |
| Cluster-261.0 | 1.4445 | + | Prot_49332 | 0.0860081 | _ | negative |
| Cluster-4742.0 | 1.4006 | + | Prot_13775 | 0.432671549 | _ | negative |
| Cluster-5849.0 | 0.047032 | _ | Prot_50073 | 0.10371135 | _ | positive |
| Cluster-1681.0 | 0.40602 | _ | Prot_26531 | 0.255607071 | _ | positive |
| Cluster-1790.0 | 2.2665 | _ | Prot_7387 | 0.385772463 | _ | positive |
| Cluster-11498.0 | 0.85979 | + | Prot_5186 | 0.055015287 | _ | negative |
| Cluster-12390.0 | 1.6243 | _ | Prot_29383 | 0.109430377 | + | negative |
| Cluster-564.0 | 3.8014 | _ | Prot_1328 | 0.219933107 | _ | positive |
| Cluster-162.0 | 1.8775 | + | Prot_6238 | 0.300631541 | _ | negative |
| Cluster-210.0 | 2.6831 | + | Prot_49211 | 0.268917819 | + | positive |
| Cluster-410.0 | 1.876 | + | Prot_49675 | 0.11281497 | _ | negative |
| Cluster-9497.0 | 0.52497 | + | Prot_51078 | -0.141189106 | _ | negative |

**Table S7** Differential metabolites between the AMF-inoculated group and the control group.

| **ID** | **Name** | **Formula** | **RT[min]** | **Mass-to-charge ratio[m/z]** | **Pvalue** | **VIP** | **Up/Down** |
| --- | --- | --- | --- | --- | --- | --- | --- |
| Com_515_pos | 13,14-dihydro-15-keto-tetranor Prostaglandin E2 | C_16_ H_26_ O_5_ | 7.311 | 321.16949 | 0.000423747 | 1.790094099 | down |
| Com_3177_pos | Neocnidilide | C_12_ H_18_ O_2_ | 6.695 | 195.13808 | 0.000575444 | 1.757446229 | up |
| Com_6012_pos | Desmethylclozapine | C_17_ H_17_ Cl N_4_ | 4.953 | 157.06726 | 0.001007235 | 1.731873248 | down |
| Com_2936_pos | 7α-Hydroxytestosterone | C_19_ H_28_ O_3_ | 7.232 | 305.20877 | 0.001392062 | 1.709859372 | down |
| Com_68_pos | D-threo-Isocitric acid | C_6_ H_8_ O_7_ | 2.108 | 193.03444 | 0.001400881 | 1.738914376 | up |
| Com_5839_neg | Parthenolide | C_15_ H_20_ O_3_ | 6.545 | 247.13422 | 0.001433255 | 1.730928604 | down |
| Com_5394_pos | 1-(2-furyl)pentane-1,4-dione | C_9_ H_10_ O_3_ | 6.726 | 189.0519 | 0.001867395 | 1.731517123 | up |
| Com_8245_neg | 3-Amino-4-methylpentanoic acid | C_6_ H_13_ NO_2_ | 5.569 | 130.08752 | 0.001993563 | 1.753095209 | down |
| Com_3932_pos | 3-(dimethylamino)-1-[4-(phenylsulfonyl)phenyl]prop-2-en-1-one | C_17_ H_17_ NO_3_ S | 2.181 | 316.10001 | 0.002179923 | 1.668165399 | up |
| Com_9548_pos | 4-Dimethylaminobenzaldehyde | C_9_ H_11_ NO | 4.981 | 150.09198 | 0.002418809 | 1.711524423 | down |
| Com_1875_neg | N-Acetyl-D-alloisoleucine | C_8_ H_15_ NO_3_ | 5.593 | 172.09801 | 0.002520933 | 1.648090627 | down |
| Com_3640_pos | 1-(4-benzylpiperazino)-2-(pyridin-2-ylamino)propan-1-one | C_19_ H_24_ N_4_O | 7.225 | 363.15652 | 0.002566092 | 1.768878913 | down |
| Com_6350_neg | Porphobilinogen | C_10_ H_14_ N_2_ O_4_ | 4.906 | 225.0883 | 0.002589786 | 1.703258765 | up |
| Com_405_neg | N-Caffeoylagmatine | C_14_ H_20_ N_4_ O_3_ | 6.142 | 291.14493 | 0.002943722 | 1.630610095 | up |
| Com_10188_pos | Ganoderic acid C6 | C_30_ H_42_ O_8_ | 8.038 | 531.29462 | 0.003030222 | 1.733534671 | down |
| Com_2473_neg | Threonine | C_4_ H_9_ NO_3_ | 1.282 | 118.05085 | 0.003066867 | 1.729044461 | up |
| Com_4802_pos | Lactucin | C_15_ H_16_ O_5_ | 5.295 | 277.10721 | 0.003073484 | 1.631693887 | down |
| Com_771_pos | 4-methyl-5-oxo-2-pentyl-2,5-dihydrofuran-3-carboxylic acid | C_11_ H_16_ O_4_ | 7.489 | 213.11211 | 0.003081813 | 1.686218635 | down |
| Com_73_neg | (±)9-HpODE | C_18_ H_32_ O_4_ | 7.406 | 311.22287 | 0.003168065 | 1.639182481 | up |
| Com_6232_pos | (+)-Catechin | C_15_ H_14_ O_6_ | 5.452 | 291.08539 | 0.003275129 | 1.780997405 | down |
| Com_1483_pos | 4-methoxy-6-(prop-2-en-1-yl)-2H-1,3-benzodioxole | C_11_ H_12_ O_3_ | 6.979 | 193.08592 | 0.003399417 | 1.641447422 | down |
| Com_2856_pos | 5-nitrothiophene-3-carbaldehyde 3-(2-pyridyl)hydrazone | C_10_ H_8_ N_4_ O_2_ S | 1.083 | 249.04533 | 0.003492187 | 1.648393313 | up |
| Com_421_pos | Mogroside III | C_48_ H_82_ O_19_ | 5.652 | 963.55286 | 0.003700955 | 1.686129491 | up |
| Com_763_pos | 2,3-Dinor-8-epi-prostaglandin F2α | C_18_ H_30_ O_5_ | 6.633 | 349.19824 | 0.003797489 | 1.643200782 | up |
| Com_4510_neg | Sibiricose A6 | C_23_ H_32_ O_15_ | 5.307 | 547.16803 | 0.003869355 | 1.676724333 | down |
| Com_8321_pos | Schisanhenol | C_23_ H_30_ O_6_ | 7.495 | 403.21054 | 0.003903843 | 1.649948518 | down |
| Com_915_pos | 6-(3-hydroxybutan-2-yl)-5-(hydroxymethyl)-4-methoxy-2H-pyran-2-one | C_11_ H_16_ O_5_ | 7.222 | 211.09639 | 0.004057363 | 1.685896399 | up |
| Com_2322_neg | 1,5,8-Trihydroxy-9-oxo-9H-xanthen-3-yl beta-D-glucopyranoside | C_19_ H_18_ O_11_ | 1.493 | 421.07547 | 0.004201211 | 1.780932605 | up |
| Com_1582_pos | Bilobalide | C_15_ H_18_ O_8_ | 5.38 | 327.10748 | 0.004336101 | 1.592243418 | down |
| Com_3652_pos | N-Acetylneuraminic acid | C_11_ H_19_ NO_9_ | 1.348 | 310.1131 | 0.004757635 | 1.675866922 | down |
| Com_2834_pos | Diacetoxyscirpenol | C_19_ H_26_ O_7_ | 5.764 | 367.17249 | 0.005120561 | 1.723766676 | up |
| Com_3208_pos | Dihydrothymine | C_5_ H_8_ N_2_O_2_ | 1.331 | 129.0659 | 0.00532034 | 1.623821255 | down |
| Com_202_pos | Nicotinic acid | C_6_ H_5_ NO_2_ | 1.37 | 124.03936 | 0.005431145 | 1.782330321 | up |
| Com_9979_neg | Puerarin | C_21_ H_20_ O_9_ | 5.494 | 415.10883 | 0.005680921 | 1.617306289 | down |
| Com_270_pos | Ginsenoside Rb1 | C_54_ H_92_ O_23_ | 6.811 | 1109.60901 | 0.005685057 | 1.574079089 | up |
| Com_4134_pos | Limonin | C_26_ H_30_ O_8_ | 6.524 | 489.20956 | 0.005703924 | 1.629907997 | down |
| Com_187_pos | Rubescensin A | C_20_ H_28_ O_6_ | 7.216 | 365.19446 | 0.00586154 | 1.685273118 | up |
| Com_1596_pos | p-Anisaldehyde | C_8_ H_8_ O_2_ | 6.926 | 137.05966 | 0.005954225 | 1.582646435 | down |
| Com_129_pos | 9-Oxo-ODE | C_18_ H_30_ O_3_ | 4.719 | 295.2265 | 0.005966197 | 1.56204898 | up |
| Com_5231_pos | Guaiacol | C_7_ H_8_ O_2_ | 5.539 | 125.05972 | 0.006054583 | 1.558970381 | down |
| Com_5755_pos | N1-phenethyl-3-(2-thienyl)acrylamide | C_15_ H_15_ NO S | 7.446 | 258.09503 | 0.006169821 | 1.595970783 | down |
| Com_9684_neg | Verbascoside | C_29_ H_36_ O_15_ | 5.421 | 623.19965 | 0.006284457 | 1.599270529 | up |
| Com_5348_neg | Raddeanin A | C_47_ H_76_ O_16_ | 5.425 | 895.50739 | 0.0063834 | 1.620213252 | down |
| Com_3407_pos | 2,4-Dimethylbenzaldehyde | C_9_ H_10_ O | 7.595 | 135.08049 | 0.007153709 | 1.555409848 | up |
| Com_1992_pos | alpha-Asarone | C_12_ H_16_ O_3_ | 5.477 | 209.11722 | 0.007157523 | 1.562952168 | down |
| Com_392_pos | Roburic acid | C_30_ H_48_ O_2_ | 7.047 | 441.37265 | 0.007182595 | 1.539866991 | down |
| Com_5006_pos | Aloesin | C_19_ H_22_ O_9_ | 1.082 | 395.1337 | 0.007227078 | 1.566815945 | up |
| Com_10673_pos | Guan-fu base A | C_24_ H_31_ NO_6_ | 5.464 | 430.22256 | 0.007564963 | 1.546538302 | down |
| Com_3997_pos | 6-(Methylthio)purine | C_6_ H_6_ N_4_ S | 7.417 | 189.02162 | 0.007632426 | 1.627435807 | up |
| Com_6497_pos | 1,7-bis(3,4-dihydroxyphenyl)heptan-3-one | C_19_ H_22_ O_5_ | 1.483 | 313.14352 | 0.007912183 | 1.534537095 | up |
| Com_1154_pos | Avocadyne 1-acetate | C_19_ H_34_ O_4_ | 1.393 | 309.2421 | 0.0085838 | 1.560901078 | down |
| Com_5035_pos | Berberine | C_20_ H_18_ NO_4_ | 4.902 | 337.13251 | 0.009051612 | 1.551099063 | up |
| Com_3619_pos | (5-L-Glutamyl)-L-amino acid | C_8_ H_14_ N_2_ O_5_ | 5.708 | 219.09744 | 0.009176678 | 1.564832706 | down |
| Com_9218_pos | 1-(3,4-dimethoxyphenyl)ethan-1-one oxime | C_10_ H_13_ NO_3_ | 8.933 | 196.09698 | 0.009523353 | 1.529968843 | up |
| Com_748_pos | T-2 Triol | C_20_ H_30_ O_7_ | 6.338 | 383.20599 | 0.00956874 | 1.521081968 | down |
| Com_1513_pos | Palmitoleic Acid | C_16_ H_30_ O_2_ | 6.259 | 277.21597 | 0.009602456 | 1.521832172 | down |
| Com_10217_pos | Artemisinin | C_15_ H_22_ O_5_ | 2.273 | 283.1525 | 0.010477264 | 1.602508277 | up |
| Com_4288_pos | olivil | C_20_ H_24_ O_7_ | 6.045 | 377.15833 | 0.010478475 | 1.529957381 | down |
| Com_2229_neg | Uridine | C_9_ H_12_ N_2_ O_6_ | 5.835 | 243.06224 | 0.010660657 | 1.521393918 | up |
| Com_1484_neg | 2-Methylbutyl beta-D-glucopyranoside | C_11_ H_22_ O_6_ | 5.544 | 249.13426 | 0.010860635 | 1.506879888 | down |
| Com_2324_pos | Notoginsenoside R1 | C_47_ H_80_ O_18_ | 1.537 | 933.53912 | 0.010943549 | 1.521155823 | up |
| Com_4011_pos | N1-(2,6-dimorpholino-3-pyridyl)-4-methylbenzamide | C_21_ H_26_ N_4_O_3_ | 4.886 | 383.20551 | 0.010958886 | 1.506223467 | down |
| Com_62_neg | Glutaconic acid | C_5_ H_6_ O_4_ | 1.495 | 129.01947 | 0.011036273 | 1.668312043 | up |
| Com_5360_neg | Suberic acid | C_8_ H_14_ O_4_ | 7.689 | 173.08183 | 0.012022969 | 1.495122876 | down |
| Com_2992_neg | N-Acetyl-α-D-glucosamine 1-phosphate | C_8_ H_16_ NO_9_P | 9.332 | 300.0491 | 0.012119975 | 1.482744624 | down |
| Com_697_pos | Testosterone undecanoate | C_30_ H_48_ O_3_ | 5.703 | 457.36691 | 0.012183914 | 1.478755017 | up |
| Com_9471_pos | Palmatine | C_21_ H_22_ NO_4_ | 9.213 | 353.16147 | 0.012515396 | 1.473297832 | up |
| Com_6635_pos | Hecogenin | C_27_ H_42_ O_4_ | 4.816 | 431.31522 | 0.012792181 | 1.529637304 | down |
| Com_864_pos | Panaxatriol | C_30_ H_52_ O_4_ | 5.653 | 477.39377 | 0.013203805 | 1.487881746 | down |
| Com_110_pos | 2-{(3S)-1-[4-(Trifluoromethyl)benzyl]-3-pyrrolidinyl}-1H-benzimidazole | C_19_ _H18_ F_3_ N_3_ | 4.995 | 346.15109 | 0.013587667 | 1.52175731 | up |
| Com_4975_pos | N-Acetylornithine | C_7_ H_14_ N_2_O_3_ | 8.413 | 175.10655 | 0.013715198 | 1.481145874 | up |
| Com_9916_pos | Orcinol glucoside | C_13_ H_18_ O_7_ | 1.973 | 287.11154 | 0.014009496 | 1.54961588 | down |
| Com_11350_pos | Cucurbitacin I | C_30_ H_42_ O_7_ | 2.115 | 515.29877 | 0.014200491 | 1.456739163 | up |
| Com_1740_pos | 4-chloro-5-morpholino-2-quinoxalin-2-ylpyridazin-3(2H)-one | C_16_ H_14_ ClN_5_ O_2_ | 6.831 | 344.09082 | 0.014357663 | 1.52156265 | down |
| Com_6138_pos | Kinetin | C_10_ H_9_ N_5_O | 6.912 | 238.07074 | 0.014702255 | 1.53816949 | down |
| Com_5350_pos | Schizandrol A | C_24_ H_32_ O_7_ | 1.398 | 450.24829 | 0.014973336 | 1.503384591 | up |
| Com_1533_pos | 6 β-Hydroxycortisol | C_21_ H_30_ O_6_ | 5.266 | 379.20898 | 0.014998037 | 1.624323127 | down |
| Com_3871_pos | Methylimidazoleacetic acid | C_6_ H_8_ N_2_ O_2_ | 5.393 | 141.06575 | 0.015041054 | 1.463012958 | down |
| Com_2135_neg | Ajugol | C_15_ H_24_ O_9_ | 2.733 | 347.13513 | 0.015165232 | 1.460488064 | up |
| Com_441_pos | Veratrole | C_8_ H_10_ O_2_ | 5.27 | 139.07539 | 0.015311151 | 1.476738931 | down |
| Com_184_pos | L-Threonic acid-1,4-lactone | C_4_ H_6_ O_4_ | 5.376 | 119.03547 | 0.015738878 | 1.461002499 | down |
| Com_1817_pos | Isosinensetin | C_20_ H_20_ O_7_ | 5.764 | 373.12762 | 0.015779134 | 1.520434861 | up |
| Com_1669_pos | Sarracenin | C_11_ H_14_ O_5_ | 5.346 | 227.09138 | 0.01589723 | 1.456599587 | down |
| Com_6936_pos | N-{5-[(dimethylamino)sulfonyl]-2-methylphenyl}cyclohexanecarboxamide | C_16_ H_24_ N_2_ O_3_S | 4.94 | 325.16336 | 0.016258151 | 1.434650168 | up |
| Com_1517_pos | 5-phenyl-2,3-dihydro-1H-1,4-benzodiazepin-2-one | C_15_ H_12_ N_2_ O | 6.577 | 259.08124 | 0.016307904 | 1.474259558 | down |
| Com_4191_neg | 3,8,9-trihydroxy-10-propyl-3,4,5,8,9,10-hexahydro-2H-oxecin-2-one | C_12_ H_20_ O_5_ | 7.955 | 289.12958 | 0.016654985 | 1.47300916 | down |
| Com_269_pos | TKK | C_16_ H_33_ N_5_O_5_ | 6.591 | 376.25906 | 0.016753266 | 1.602990781 | up |
| Com_5184_neg | Jujuboside B | C_52_ H_84_ O_21_ | 5.73 | 1043.54504 | 0.016759363 | 1.451440318 | down |
| Com_1454_neg | Artesunate | C_19_ H_28_ O_8_ | 5.14 | 383.17062 | 0.016938565 | 1.454866995 | down |
| Com_2678_pos | pteroside A | C_21_ H_30_ O_8_ | 5.525 | 411.20111 | 0.01701715 | 1.46561346 | up |
| Com_11768_pos | 5-Ethyl-2'-deoxyuridine | C_11_ H_16_ N_2_ O_5_ | 9.01 | 257.11313 | 0.018496354 | 1.473836435 | down |
| Com_2337_pos | 3-(4-hydroxy-3-methoxyphenyl) propanoic acid | C_10_ H_12_ O_4_ | 7.226 | 219.06509 | 0.018704306 | 1.447366249 | down |
| Com_6220_neg | LPC 16:1 | C_24_ H_48_ NO_7_ P | 5.332 | 538.31549 | 0.018780141 | 1.436564146 | up |
| Com_4444_pos | N', N''-p-Coumaroyl, Feruloylspermidine | C_26_ H_33_ N_3_ O_5_ | 5.599 | 468.24927 | 0.019331774 | 1.41428992 | down |
| Com_7448_neg | Neonuezhenide | C_31_ H_42_ O_18_ | 5.051 | 701.23212 | 0.019346159 | 1.414568996 | up |
| Com_3728_pos | H-Gly-Pro-OH | C_7_ H_12_ N_2_ O_3_ | 7.421 | 173.09131 | 0.019393772 | 1.500084321 | up |
| Com_7190_pos | 5,6-dimethyl-3-[5-(trifluoromethyl)pyridin-2-yl]-1,2,4-triazine | C_11_ H_9_ F_3_ N_4_ | 5.352 | 255.08481 | 0.019729404 | 1.424554999 | down |
| Com_1068_neg | 5,6-Dehydroginsenoside Rd | C_48_ H_80_ O_18_ | 5.599 | 943.52875 | 0.019731954 | 1.681312686 | down |
| Com_1915_neg | Protectin D1 | C_22_ H_32_ O_4_ | 5.051 | 349.18652 | 0.020045821 | 1.434443099 | down |
| Com_5753_neg | LPE 16:0 | C_21_ H_44_ NO_7_ P | 7.421 | 452.27853 | 0.021083602 | 1.479566923 | up |
| Com_9252_pos | Tenuifolin | C_36_ H_56_ O_12_ | 5.352 | 681.38641 | 0.021434192 | 1.48575481 | down |
| Com_9524_pos | 7-Ethylcamptothecin | C_22_ _H20_ N_2_O_4_ | 9.337 | 377.15143 | 0.021521857 | 1.4607434 | up |
| Com_4713_neg | Thymidine | C_10_ H_14_ _N2_O_5_ | 7.4 | 241.08315 | 0.021763336 | 1.447569248 | down |
| Com_12126_pos | Tigloylgomisin H | C_28_ H_36_ O_8_ | 1.24 | 501.24744 | 0.021995355 | 1.541764491 | up |
| Com_5433_pos | N-Acetylglucosamine | C_8_ H_15_ NO_6_ | 4.831 | 222.09686 | 0.022657507 | 1.432307596 | down |
| Com_2058_neg | 17(S)-HpDHA | C_22_ H_32_ O_4_ | 7.852 | 395.19235 | 0.022709023 | 1.413451539 | up |
| Com_2311_pos | (+)-Dihydrojasmonic acid | C_12_ H_20_ O_3_ | 1.344 | 213.14844 | 0.023065752 | 1.441799045 | down |
| Com_404_pos | L-Aspartic acid | C_4_ H_7_ NO_4_ | 5.358 | 134.04471 | 0.023664933 | 1.487682126 | up |
| Com_1022_pos | Phosphocholine | _C5_ H_14_ NO4P | 6.896 | 184.07054 | 0.023985508 | 1.585382967 | down |
| Com_4347_neg | N7-Methylguanosine | C_11_ H_17_ N_5_O_5_ | 1.372 | 298.11435 | 0.024296259 | 1.63574331 | up |
| Com_3087_pos | Riboflavin | C_17_ H_20_ N_4_O_6_ | 1.31 | 377.14551 | 0.02437802 | 1.408607853 | up |
| Com_6001_pos | Boc-beta-cyano-L-alanine | C_9_ H_14_ N_2_O_4_ | 1.284 | 215.10251 | 0.02482092 | 1.412585626 | down |
| Com_5919_pos | 7-methylguanine | C_6_ H_7_ N_5_ O | 5.376 | 166.07278 | 0.024992826 | 1.477289242 | up |
| Com_1943_neg | Soyasaponin I | C_48_ H_78_ O_18_ | 4.845 | 941.51288 | 0.025335316 | 1.479620264 | down |
| Com_408_neg | D-Mannose 6-phosphate | C_6_ H_13_ O9P | 4.81 | 213.01712 | 0.025818289 | 1.582148482 | down |
| Com_130_pos | Ginsenoside Rg2 | C_42_ H_72_ O_13_ | 7.841 | 767.49371 | 0.025886721 | 1.503590251 | up |
| Com_2315_neg | trans-zeatin 9-O-glucoside | C_16_ H_23_ N_5_O_6_ | 1.487 | 380.15652 | 0.026438988 | 1.38565258 | down |
| Com_10314_pos | Lithospermoside | C_14_ H_19_ NO_8_ | 5.953 | 330.11823 | 0.026665898 | 1.371936019 | down |
| Com_258_pos | Asparagine | C_4_ H_8_ N_2_O_3_ | 5.017 | 133.0607 | 0.026731216 | 1.389186817 | up |
| Com_1321_pos | DQH | C_15_ H_22_ N_6_O_7_ | 4.689 | 399.16251 | 0.026748529 | 1.403679551 | up |
| Com_82_pos | Adenosine | C_10_ H_13_ N_5_O_4_ | 1.372 | 268.1037 | 0.027277324 | 1.453942323 | down |
| Com_1488_pos | Eicosapentaenoic acid | C_20_ H_30_ O_2_ | 5.468 | 309.2424 | 0.027320041 | 1.392109895 | up |
| Com_2644_pos | cyclohexyl{4-[4-nitro-2-(1H-pyrrol-1-yl)phenyl]piperazino}methanone | C_21_ H_26_ N_4_O_3_ | 2.506 | 383.20367 | 0.028179541 | 1.353332344 | up |
| Com_4917_neg | Phenylglyoxylic acid | C_8_ H_6_ O_3_ | 8.092 | 149.0242 | 0.028704756 | 1.418872227 | down |
| Com_3637_neg | Phenobarbital | C_12_ H_12_ N_2_O_3_ | 6.62 | 231.07764 | 0.029063435 | 1.376719542 | down |
| Com_4421_pos | Dehydroepiandrosterone (DHEA) | C_19_ H_28_ O_2_ | 5.227 | 267.23184 | 0.029136544 | 1.360004746 | up |
| Com_2999_pos | 4-(2,3-dihydro-1,4-benzodioxin-6-yl)butanoic acid | C_12_ H_14_ O_4_ | 4.878 | 263.09128 | 0.029523032 | 1.402744649 | down |
| Com_1164_neg | 9-HpOTrE | C_18_ H_30_ O_4_ | 8.16 | 309.20712 | 0.029792127 | 1.386683927 | up |
| Com_6963_neg | Xanthurenic acid O-hexoside | C_16_ H_17_ NO_9_ | 5.263 | 366.08316 | 0.029880383 | 1.463343964 | down |
| Com_4200_pos | Sarsasapogenin | _C27_ H_44_ O_3_ | 7.133 | 417.33563 | 0.030096583 | 1.372232972 | up |
| Com_2235_pos | Enterodiol | C_18_ H_22_ O_4_ | 4.639 | 303.15894 | 0.030116185 | 1.36952256 | down |
| Com_1249_pos | 1,2-dihydroxyheptadec-16-yn-4-yl acetate | C_19_ H_34_ O_4_ | 7.408 | 349.23453 | 0.031151641 | 1.329444836 | up |
| Com_3957_neg | Methyl gallate | C_8_ H_8_ O_5_ | 5.598 | 183.02994 | 0.031592896 | 1.430447149 | down |
| Com_5264_neg | Salvinorin A | C_23_ H_28_ O_8_ | 8.004 | 431.17468 | 0.032643774 | 1.334122987 | down |
| Com_3857_pos | Dacarbazine | C_6_ H_10_ N_6_ O | 5.078 | 183.09927 | 0.032975669 | 1.420079607 | up |
| Com_3962_neg | Angoroside C | C_36_ H_48_ O_19_ | 6.143 | 783.27203 | 0.033877446 | 1.38544464 | down |
| Com_9195_pos | Cinobufagin | C_26_ H_34_ O_6_ | 5.298 | 443.24265 | 0.034818836 | 1.376247601 | down |
| Com_5597_pos | Glutathione | C_10_ H_17_ N_3_ O_6_ S | 5.528 | 308.09128 | 0.034869851 | 1.357549489 | up |
| Com_1709_neg | Triethyl citrate | C_12_ H_20_ O_7_ | 8.337 | 275.11392 | 0.036275348 | 1.361728685 | down |
| Com_5922_pos | Pomolic acid beta-D-glucopyranosyl ester | C_36_ H_58_ O_9_ | 5.645 | 635.41516 | 0.037827877 | 1.337719042 | down |
| Com_1694_neg | Madecassoside | C_48_ H_78_ O_20_ | 5.058 | 973.50128 | 0.038002858 | 1.294783142 | up |
| Com_5342_pos | Asp-phe | C_13_ H_16_ N_2_ O_5_ | 6.438 | 281.11316 | 0.038548338 | 1.292625496 | down |
| Com_4574_pos | TQH | C_15_ H_24_ N_6_O_6_ | 8.478 | 385.18295 | 0.039017049 | 1.377808249 | down |
| Com_290_neg | 1,2,3-cyclopropanetricarboxylic acid | C_6_ H_6_ O_6_ | 5.242 | 173.00906 | 0.039254889 | 1.348568781 | up |
| Com_3181_pos | Senkyunolide A | C_12_ H_16_ O_2_ | 5.253 | 193.1221 | 0.039477131 | 1.315748843 | up |
| Com_3134_pos | 7-hydroxy-4-[(2-pyridylthio)methyl]-2H-chromen-2-one | C_15_ H_11_ N O_3_ S | 2.122 | 286.05328 | 0.039741379 | 1.371927358 | down |
| Com_7409_neg | Ginkgolide C | C_20_ H_24_ O_11_ | 5.609 | 439.12546 | 0.040265915 | 1.309572154 | down |
| Com_1702_neg | Bergenin | C_14_ H_16_ O_9_ | 1.528 | 327.07217 | 0.040645781 | 1.313601506 | up |
| Com_5237_neg | 4,5-DCQA Isochlorogenic acid C | C_25_ H_24_ O_12_ | 5.728 | 515.12152 | 0.040701359 | 1.294591633 | down |
| Com_537_neg | 20(R)-Ginsenoside Rh1 | C_36_ H_62_ O_9_ | 5.213 | 683.4386 | 0.040735886 | 1.362724845 | down |
| Com_2911_pos | Naringin Dihydrochalcone | C_27_ H_34_ O_14_ | 4.978 | 583.19977 | 0.04084874 | 1.279227834 | up |
| Com_4044_neg | N-Acetyl-DL-valine | C_7_ H_13_ NO_3_ | 6.65 | 158.08209 | 0.040994962 | 1.323210176 | down |
| Com_951_pos | 2,5-bis(4-hydroxy-3-methoxyphenyl)-3,4-dimethyloxolan-3-ol | C_20_ H_24_ O_6_ | 6.04 | 343.1514 | 0.040997499 | 1.476031908 | down |
| Com_2872_pos | Jasmonic acid | C_12_ H_18_ O_3_ | 5.259 | 211.13269 | 0.041119962 | 1.302397907 | up |
| Com_609_neg | methyl oxo pentanoate | C_6_ H_10_ O_3_ | 7.123 | 129.05556 | 0.041131109 | 1.293710158 | up |
| Com_3911_neg | (S)-Zearalanone | C_18_ H_24_ O_5_ | 6.699 | 319.15543 | 0.042287775 | 1.331869414 | down |
| Com_2952_pos | N1-(5-methylisoxazol-3-yl)-2-morpholinoacetamide | C_10_ H_15_ N_3_O_3_ | 5.795 | 264.07031 | 0.042381097 | 1.330826927 | down |
| Com_7973_pos | 13-α-(21)-Epoxyeurycomanone | C_20_ H_24_ O_10_ | 7.074 | 425.14697 | 0.042432786 | 1.508082271 | up |
| Com_5595_pos | 3-[(5-nitropyridin-2-yl)oxy]-1H-indazole | C_12_ H_8_ N_4_ O_3_ | 1.399 | 235.08311 | 0.042515456 | 1.329159954 | down |
| Com_2651_neg | Neomangiferin | C_25_ H_28_ O_16_ | 7.915 | 583.12848 | 0.042708856 | 1.28398042 | down |
| Com_566_neg | alpha-Zearalanol | C_18_ H_26_ O_5_ | 5.092 | 367.1763 | 0.042936408 | 1.323216101 | up |
| Com_231_pos | Heroin-d3 | C_21_ H_2_0 [2]H_3_ NO_5_ | 1.492 | 373.18518 | 0.043063505 | 1.30077843 | down |
| Com_1574_pos | Eupalinolide A | C_24_ H_30_ O_9_ | 5.693 | 463.19794 | 0.043896481 | 1.329036992 | down |
| Com_5502_neg | 4-Methylbenzotriazole | C_7_ H_7_ N_3_ | 5.62 | 132.05658 | 0.044060248 | 1.343986647 | up |
| Com_1479_pos | Abscisic acid glucose ester | C_21_ H_30_ O_9_ | 5.715 | 427.19513 | 0.044109683 | 1.309665534 | up |
| Com_2869_pos | L-5-Hydroxytryptophan | C_11_ H_12_ N_2_O_3_ | 5.185 | 221.0921 | 0.044552611 | 1.273163 | down |
| Com_6617_pos | Muramic acid | C_9_ H_17_ NO_7_ | 6.491 | 252.10754 | 0.045847907 | 1.330268145 | down |
| Com_661_pos | 10-Deacetylbaccatin-III | C_29_ H_36_ O_10_ | 4.939 | 545.23773 | 0.046030617 | 1.31117162 | down |
| Com_7305_pos | Fraxinellone | C_14_ H_16_ O_3_ | 1.35 | 251.12773 | 0.046110237 | 1.258465823 | up |
| Com_7572_pos | Decursin | C_19_ H_2_O O_5_ | 7.256 | 329.13785 | 0.046374364 | 1.263906768 | down |
| Com_4798_neg | Asperulosid | C_18_ H_22_ O_11_ | 5.926 | 413.10901 | 0.046507446 | 1.283712536 | down |
| Com_7908_pos | Pimpinellin | C_13_ H_10_ O_5_ | 5.459 | 247.05989 | 0.047125131 | 1.259537859 | up |
| Com_6661_pos | N-lactoyl-phenylalanine | C_12_ H_15_ NO_4_ | 5.419 | 238.10757 | 0.047130156 | 1.282156315 | down |
| Com_1967_pos | Sauchinone | C_20_ H_20_ O_6_ | 4.771 | 357.13339 | 0.047180386 | 1.285954437 | down |
| Com_8470_neg | Kynurenic acid O-hexside | C_16_ H_17_ NO_8_ | 5.245 | 350.08804 | 0.047310852 | 1.335851351 | down |
| Com_6178_pos | 4-cyclopropyl-6-(methylthio)-2-pyrazin-2-ylpyrimidine-5-carbonitrile | C_13 H11_ N_5_S | 5.596 | 270.07916 | 0.047650142 | 1.300736513 | up |
| Com_4573_neg | Lysopa 16:0 | C_19_ H_39_ O_7_ P | 5.262 | 409.23779 | 0.047651465 | 1.35116813 | down |
| Com_4970_neg | 2-Hydroxyisocaproic Acid | C_6_ H_12_ O_3_ | 1.854 | 131.07162 | 0.04778904 | 1.321848868 | down |
| Com_4257_neg | Poncirin | C_28_ H_34_ O_14_ | 9.29 | 593.18774 | 0.047870061 | 1.279357842 | up |
| Com_3891_pos | L-Mimosine | C_8_ H_10_ N_2_ O_4_ | 5.882 | 199.07114 | 0.048340011 | 1.278052044 | up |
| Com_4523_neg | Polygalaxanthone Ⅵ | C_23_ H_26_ O_12_ | 5.316 | 493.13596 | 0.049218364 | 1.435031047 | up |
| Com_3403_pos | Syringenone | C_17_ H_24_ O_9_ | 1.393 | 373.14932 | 0.049311874 | 1.301155586 | down |
